# Human-biased *MEIS1* expression by a promoter noncoding RNA regulates neural stem cell proliferation

**DOI:** 10.26508/lsa.202603708

**Published:** 2026-09-30

**Authors:** Hayato Uchida, Tomonori Kameda, Shota Zenno, Hideyuki Nakashima, Yuki Minato, Yuina Aritsuka, Sota Nishida, Boyang An, Mizuki Honda, Fumihiro Morishita, Kinichi Nakashima, Takuya Imamura

**Affiliations:** 1 Program of Biomedical Science, Graduate School of Integrated Sciences for Life, Hiroshima University , Higashi-Hiroshima, Japan; 2 Department of Stem Cell Biology and Medicine, Graduate School of Medical Sciences, Kyushu University, Fukuoka, Japan

## Abstract

Whereas primate-specific genes are known to drive human evolution, conserved genes also play critical roles in defining species-specific traits. This study identifies *MEIS1* as a key conserved gene with significantly higher expression in human radial glia compared with those in mice. Our functional analysis revealed that *MEIS1* knockdown (KD) in human cerebral organoids leads to premature neural differentiation. Conversely, *MEIS1* overexpression in the mouse brain significantly increases the abundance of neural progenitor cells (NPCs). By investigating a noncoding RNA (ncRNA) partner derived from the *MEIS1* promoter, we demonstrated that its KD triggers *MEIS1* down-regulation, markedly reducing NPC proliferative capacity. RNA-sequencing further indicated that *MEIS1* regulates genes involved in cholesterol biogenesis. Specifically, forced expression of *DHCR7*, a *MEIS1*-target gene, rescued both the proliferative deficits and the reduction in primary cilia length induced by *MEIS1* KD. These findings suggest that ncRNA-mediated regulatory mechanism for the conserved *MEIS1* coordinates primary cilia morphology and cholesterol metabolism. This coordination is essential for maintaining long-term NPC properties, ultimately contributing to the evolutionary expansion of the human cerebral cortex.

## Abstract (precis)

Conserved *MEIS1* drives human cortical expansion. A human-specific noncoding RNA regulates *MEIS1* to coordinate primary cilia morphology, preserving neural progenitor proliferation

## Introduction

The brains exhibit great difference structurally and functionally between rodents and primates. Expansion of the neocortex and gyrification are hallmarks of primate brain evolution. Especially the expansion of the neocortex, which in humans is the site of our cognitive abilities, is thought to be primarily due to an increase in neuron production (Herculano-Houzel, 2017). This process, along with overall neocortical size, is governed by the abundance and proliferative capacity of neural progenitor cells (NPCs) during embryonic/fetal development. NPCs can be divided into two classes: apical progenitors (APs) and basal progenitors (BPs). APs reside in the ventricular zone (VZ), are highly proliferative across mammals, and upon division can generate other APs, BPs, or, rarely, neurons (Rakic, 2003; Götz & Huttner, 2005). BPs reside in the subventricular zone (SVZ) and show great variability in their proliferative capacity across mammals. In species with a small and smooth neocortex, such as mouse, BPs exhibit a low proliferative capacity, dividing typically once to generate two neurons (Haubensak et al, 2004; Miyata et al, 2004; Noctor et al, 2004). In species with an expanded and folded neocortex, such as human, BPs are more proliferative and can undergo several cycles of proliferative divisions to generate further BPs before dividing to generate neurons (Lui et al, 2011; Florio & Huttner, 2014). A specific type of BPs, called basal (or outer) radial glia (bRG), is particularly important in this context. In mouse, bRG are very rare and have limited self-renewal capacity (Shitamukai et al, 2011; Wang et al, 2011), whereas in primates, bRG are more abundant and highly proliferative with extensive self-renewal capacity (Smart et al, 2002; Fietz et al, 2010; Hansen et al, 2010; Reillo et al, 2011; Betizeau et al, 2013). The increased proliferative capacity of bRG leads to SVZ expansion, resulting in the generation of two distinct zones: inner and outer SVZ (ISVZ and OSVZ) (Smart et al, 2002; Dehay et al, 2015). Thus, BPs, and particularly bRG, are considered the major progenitor cell type underlying the evolutionary expansion of the neocortex (Lui et al, 2011; Borrell & Reillo, 2012; Florio & Huttner, 2014).

In the search for the genomic basis underlying human neocortex expansion, researchers have focused on genes, notably human-specific genes, that are preferentially expressed in the stem and progenitor cells of the fetal human neocortex (Bystron et al, 2006; Rakic, 2009; Lui et al, 2011; Borrell & Reillo, 2012; Florio & Huttner, 2014; Dehay et al, 2015; Florio et al, 2015; Liu et al, 2017; Sousa et al, 2017; Fischer et al, 2022). Because the diversity of noncoding regions among species is rather extremely high, species differences in gene expression affected by them should also constitute the structural and functional characteristics of human neocortex. We have previously shown that promoter-associated noncoding RNAs (pancRNAs), which can specifically activate partner genes (Uesaka et al, 2014; Hamazaki et al, 2015), are generated from hundreds of non-conserved regions and that these exhibit species-pattern of transcriptomes in general (Uesaka et al, 2017). We assume that a set of evolutionary acquired human-specific pancRNAs mediates the formation of human-specific mRNA transcriptomes to alter NPC behaviors. This hypothesis is supported by our previous data showing that *TMEM25*, a human-specific pancRNA-partnered gene, accelerate the expansion of bRG (An et al, 2023).

The proliferative capacity of NPCs, which is essentially species-specific as described above, is intricately linked to specific cellular programs, including metabolic pathways and organelle dynamics. Recent studies have suggested that cholesterol, derived either from surrounding cell types or produced cell-autonomously, functions as a crucial signaling regulator in developing neural progenitors, specifically by modulating lipid raft formation and primary cilia dynamics, which are essential for transducing proliferative signals (Suzuki et al, 2020; Vona et al, 2021; Nourse et al, 2022; Park et al, 2023).

In this study, we focused on *MEIS1* as a potential key transcription factor in shaping the human NPC transcriptome. *MEIS1* is well known for its crucial role in driving neural progenitor regulation during cerebellar foliation—a process involving local progenitor proliferation and tissue folding structurally analogous to cerebral cortical gyrification (Owa et al, 2018). Moreover, previous studies using in utero electroporation in mouse embryonic cortices demonstrated that forced expression of *Meis1* promotes the proliferation of NPCs (Isogai et al, 2022). However, how *MEIS1* expression is evolutionary tuned in the human lineage and whether it contributes to the evolutionary expansion of the neocortex remain unknown. Here, we provide evidence that the human-biased *pancRNA-MEIS1* axis regulates cholesterol metabolism to sustain primary cilia formation, thereby maintaining the proliferative capacity of human NPCs. Mimicking human-type *MEIS1* expression in mouse cortices expanded the basal progenitor pool, whereas its suppression in human models compromised NPC ciliogenesis by down-regulating genes related to cholesterol biosynthesis. Our findings highlight a novel mechanism wherein the activation of a conserved gene by a species-specific noncoding RNA drives the evolutionary expansion of the human neocortex through metabolic regulation of ciliogenesis.

## Results

### *MEIS1* was highly expressed in human neocortex compared with mice

**Figure 1.**
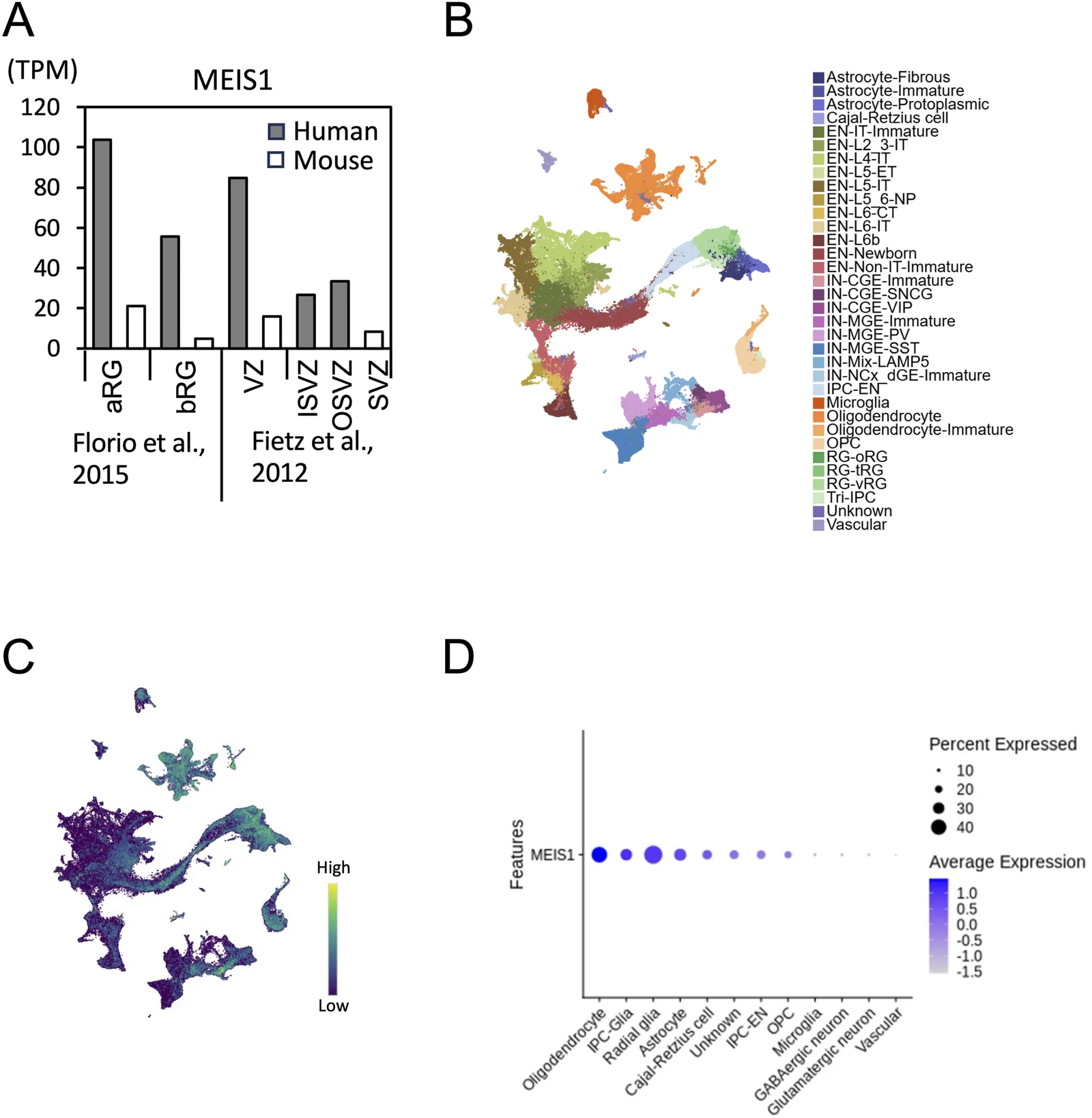
*MEIS1* was highly expressed in human neocortex compared with mice. **(A)** Expression level of *MEIS1*, as analyzed using public RNA-seq data (aRGs and bRGs: [Florio et al, 2015]: [N = 4], VZ, ISVZ, OSVZ, and SVZ: [Fietz et al, 2012]: [N = 1]). Expression levels are presented as TPM (transcripts per million; indicating the relative abundance of a specific transcript per million total transcripts). **(B, C, D)** Visualization of *MEIS1* expression in single-nucleus RNA-seq data (Wang et al, 2025) of the human fetal cerebrum ((B): Cell-type label (C): Feature Plot, (D): Dot Plot). SVZ, subventricular zone; VZ, ventricular zone; ISVZ, inner subventricular zone; OSVZ, outer subventricular zone.

To investigate species differences in *MEIS1* expression in the neocortex, two previously published RNA-seq datasets (Fietz et al, 2012; Florio et al, 2015) were analyzed. *MEIS1* expression was significantly higher in human aRG, bRG, VZ, and SVZ, which are regions enriched for neural progenitor cells (NPCs) than in those of mice (Fig 1A).

To further characterize the cell-type specificity of *MEIS1* expression, single-nucleus RNA-seq from the human fetal cortex (Wang et al, 2025) were analyzed. This analysis revealed that *MEIS1* is strongly enriched in radial glial cells (Fig 1B–D). Taken together, these results suggest that *MEIS1* may play a specialized role in regulating the behavior of human NPCs in embryonic human neocortex.

### Accelerated neuronal differentiation upon *MEIS1* knockdown

**Figure 2.**
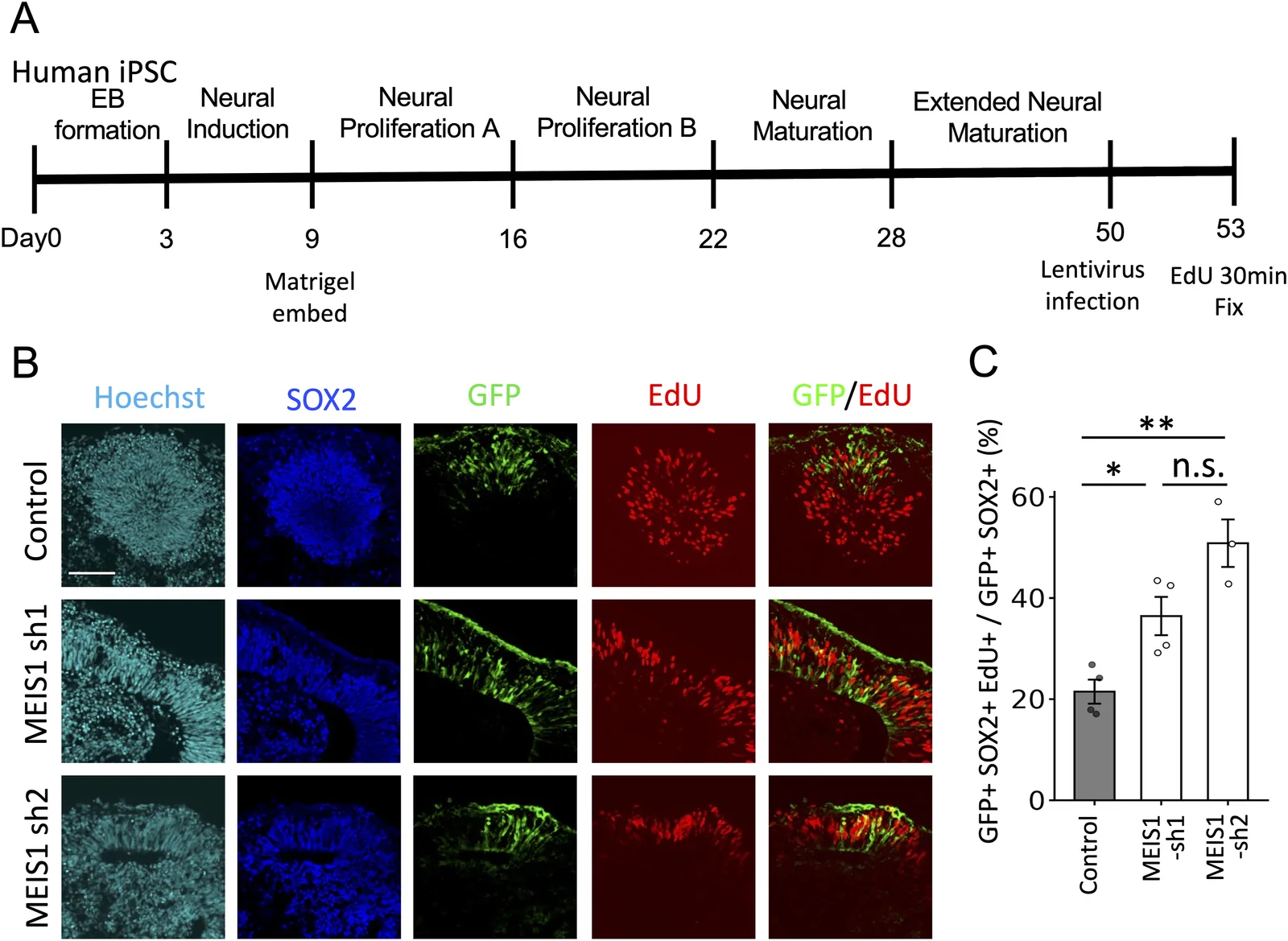
*MEIS1* KD transiently accelerated the cell cycle pregression. **(A)** Schematic protocol of cortical organoid culture and infection of lentivirus and treatment of EdU. **(B)** Representative images of cortical organoid section (Day 53) stained with antibody against SOX2 or GFP and stained with EdU and Hoechst. Scale bar = 100 μm. **(C)** Quantification of GFP+ SOX2+ EdU+/GFP+ SOX2+ (%) in (B) (n = 4 organoids for control and *MEIS1* sh1, n = 3 organoids for *MEIS1* sh2, more than three rosette per replicate). *P < 0.05, **P < 0.01, n.s., not significant. Tukey–Kramer test.

**Figure 3.**
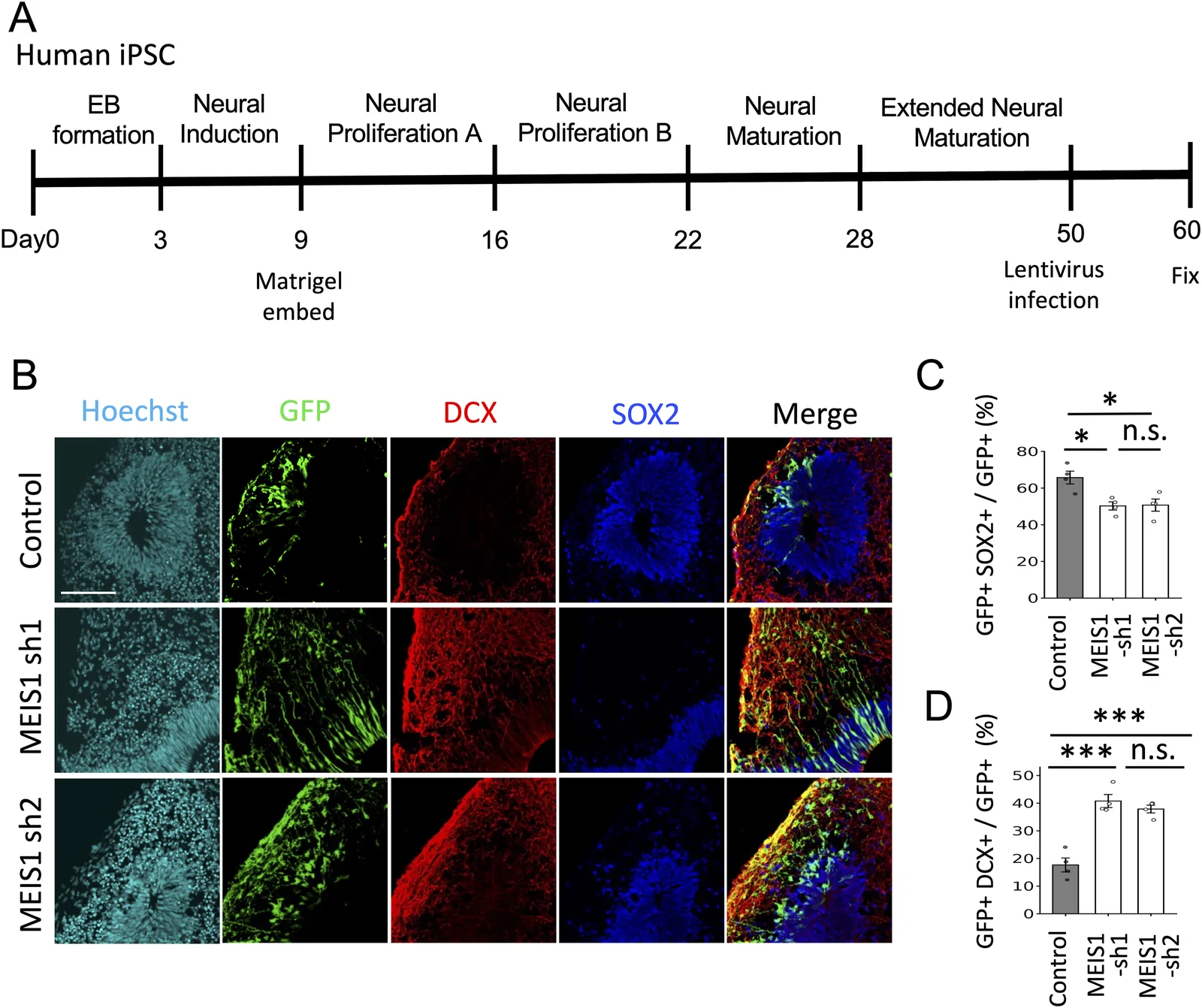
*MEIS1* KD accelerated neuronal differentiation. **(A)** Schematic protocol of cortical organoid culture and infection of lentivirus. **(B)** Representative images of cortical organoid section (Day 60) stained with antibody against SOX2, GFP, or DCX and stained with Hoechst. Scale bar = 100 μm. **(C, D)** Quantification of GFP+ SOX2+/GFP+ (%) (C) and GFP+ DCX+/GFP+ (%) (D) in (B) (n = 4 organoids for control and *MEIS1* sh1, n = 3 organoids for *MEIS1* sh2, more than three rosettes per replicate). *P < 0.05, ***P < 0.001, n.s., not significant. Tukey–Kramer test.

Brain organoids were generated from human iPSCs to mimic human cerebral development. The experimental scheme is shown in Fig 2A. When the proportion of EdU+ cells among SOX2/GFP double-positive population was examined by immunohistochemistry (Fig 2B), *MEIS1* knockdown (KD) significantly increased the proportion of EdU+ NPCs (Fig 2C). Next, immunohistochemistry was performed 10 d after the infection to examine the neural differentiation after *MEIS1* KD (Fig 3A). The proportion of SOX2+ or DCX+ cells among GFP+ cells was examined by immunohistochemistry (Fig 3B). SOX2+ cells were defined as representative of the VZ and DCX+ cells as non-VZ, with the combined total of SOX2+ and DCX+ cells accounting for more than 80% of GFP-positive cells (Fig 3C and D). Under this criterion, *MEIS1* KD showed significant decrease in the proportion of SOX2+ cells and increase in the proportion of DCX+ cells (Fig 3C and D). The increased proliferation of NPCs observed after *MEIS1* KD did not result in an expansion of the NPC pool. Indeed, 10 d after *MEIS1* KD, the proportion of SOX2+ cells significantly decreased, whereas DCX+ cells concurrently increased. These data raised the possibility that *MEIS1* KD accelerated the cell cycle transition of human NPCs, leading to their premature exhaustion and subsequent differentiation into neurons, a process reminiscent of typical mouse brain development.

### Overexpression of *MEIS1* prolonged neural progenitor cells stemness in mouse brain in vivo

**Figure 4.**
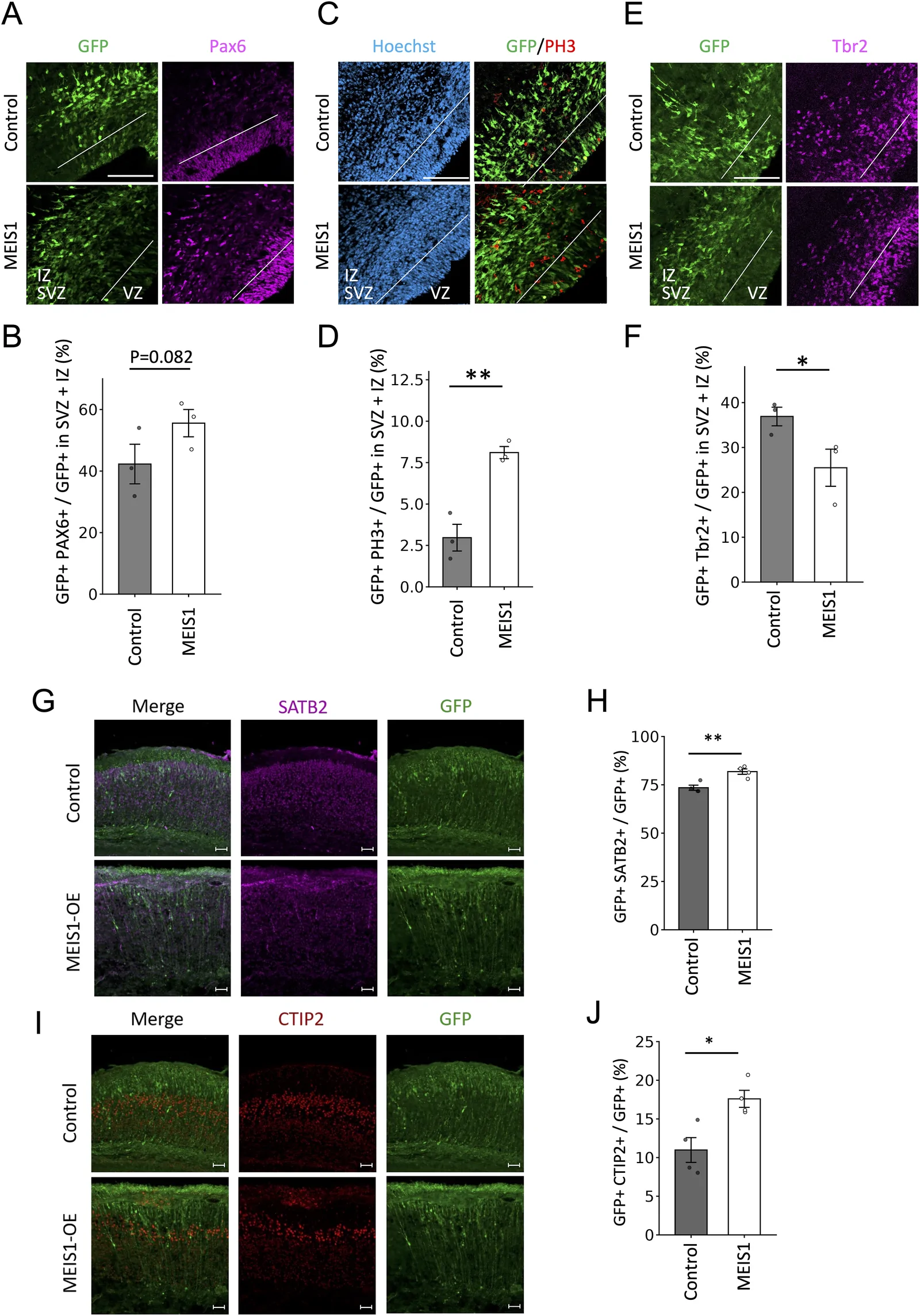
*MEIS1* overexpression prolonged NPC stemness in mouse brain in vivo. **(A, C, E)** Representative images of brain section (E15.5: 2 d after in utero electroporation) stained with antibody against GFP or PAX6 (A) and stained with antibody against GFP or PH3 and stained with Hoechst (C) and stained with antibody against GFP or TBR2 (D). Mouse embryos were electroporated in utero with plasmids expressing GFP, GFP together with *MEIS1* at E13.5 and euthanized at E15.5. Scale bar = 100 μm. **(B, D, F)** Quantification of GFP+ Pax6+/GFP+ in SVZ +IZ (%) in (A) and GFP+ PH3+/GFP+ in SVZ +IZ (%) in (B) and GFP+ Tbr2+/GFP+ in SVZ +IZ (%) in (C) (n = 4). *P < 0.05, **P < 0.01. *t* test. **(G, I)** Representative images of brain section (E18.5: 5 d after in utero electroporation) stained with antibody against GFP or SATB2 (G) and stained with antibody against GFP or CTIP2 and stained with Hoechst. **(H, J)** Quantification of GFP+ SATB2/GFP+ cells (%) in (G) and GFP+ CTIP2/GFP+ cells in (I) (n = 4). *P < 0.05, **P < 0.01. Unpaired test. Scale bar = 50 μm. IZ, intermediate zone; SVZ, subventricular zone; VZ, ventricular zone.

An experiment was conducted in which *MEIS1* was forced expressed in NPCs during mouse brain development using in utero electroporation method. When PAX6-positive cells or the mitotic marker phospho-histone H3 in the SVZ region were examined by immunohistochemistry (Fig 4A and C), forced expression of *MEIS1* increased the proportion of these markers in SVZ (Fig 4B and D). On the other hand, the proportion of intermediate progenitor marker Tbr2-positive cells decreased (Fig 4E and F). These results suggested that high expression of *MEIS1* maintained the stem cell property of NPCs, one of the important human-specific features. In addition, quantification of cortical layer markers in E18 mouse brains revealed that *MEIS1* overexpression significantly increased the proportions of both CTIP2-positive deep-layer neurons and SATB2-positive upper layer neurons (Fig 4G–J).

### *MEIS1* KD decreased NPC marker gene expression in human iPSC-derived NPCs

**Figure 5.**
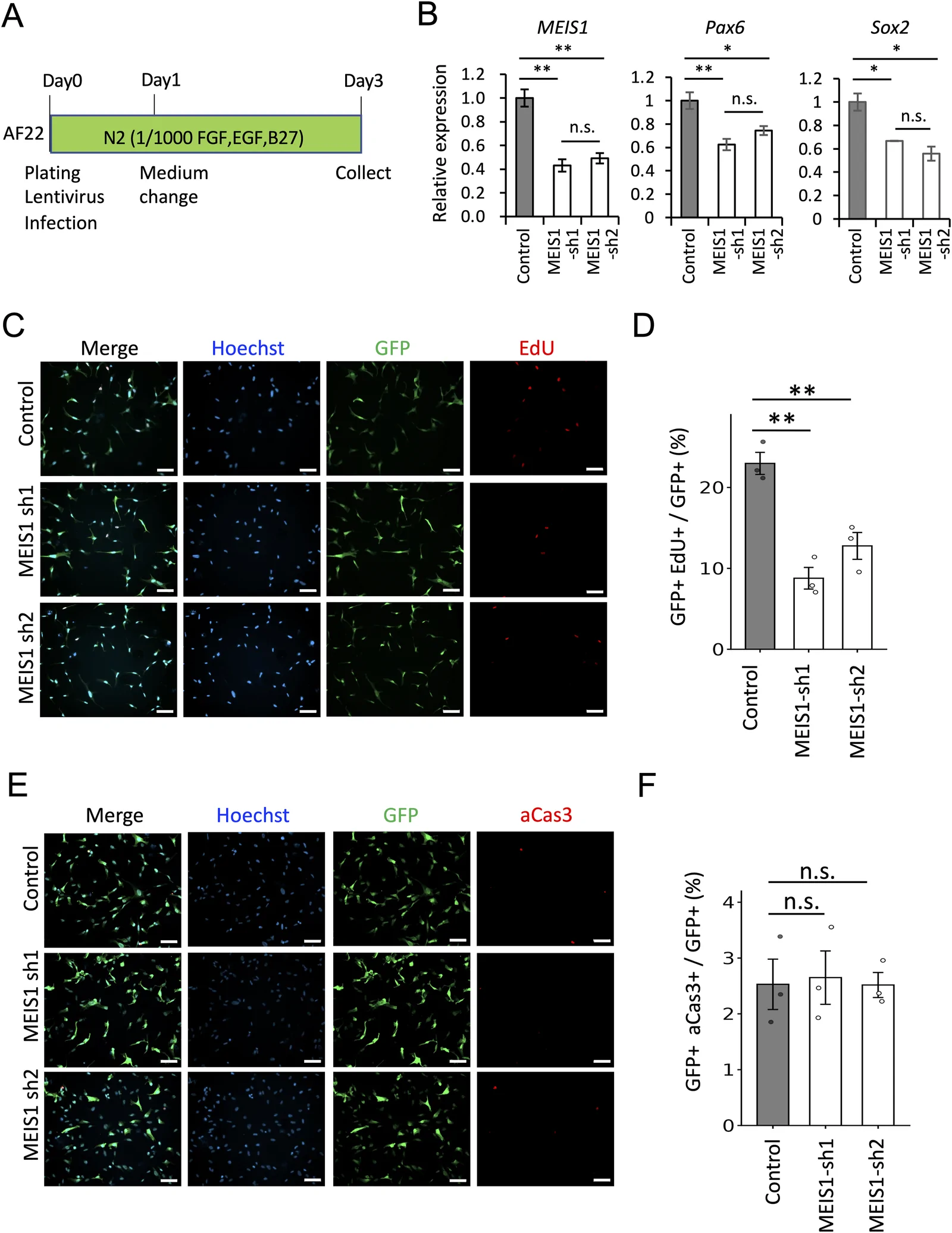
*MEIS1* KD reduced NPC marker expression and slowed cell cycle progression in human iPSC-drived NPCs. **(A)** Scheme for introducing shRNA into AF22 via lentivirus. **(B)** Quantification of shRNA KD Efficiency. **(B)** Quantification of stem cell markers SOX2 and PAX6 expression upon *MEIS1* KD. Tukey’s test, *P < 0.05, **P < 0.01, n.s. = not significant. Values in the panels indicate mean ± SEM. **(C)** Representative images of EdU uptake experiment in AF22 stained for GFP (green) and EdU (red) in control and *MEIS1* KD. Nuclei were stained with Hoechst (blue). **(D)** Quantification of EdU+GFP+ to GFP+ cells in (C). **(E)** Representative images of immunocytochemistry stained for GFP (green) and active Caspase 3 (red) in control and *MEIS1* KD. Nuclei were stained with Hoechst (blue). **(F)** Quantification of active Caspase 3+GFP+ to GFP+ cells in (E). Tukey’s test, *P < 0.05, **P < 0.01, n.s. = not significant. Values in all panels indicate mean ± SEM (C, D, E, F), n = 3. Scale bar = 100 μm.

Human iPSC-derived NPCs (AF22) were used to investigate the effects of lentivirus-based *MEIS1* KD on downstream gene expression. The experimental scheme for *MEIS1* KD is shown in Fig 5A. *MEIS1* KD in the culture system exhibited more than 50% efficiency (Fig 5B). When the expression of *PAX6*, known as a downstream gene of *MEIS1* (Owa et al, 2018), was examined, it showed statistically significant reduction by *MEIS1* KD (Fig 5B). The expression of the stem cell marker *SOX2* also showed statistically significant reduction (Fig 5B), further supporting our correct experimental setup.

**Figure S1.**
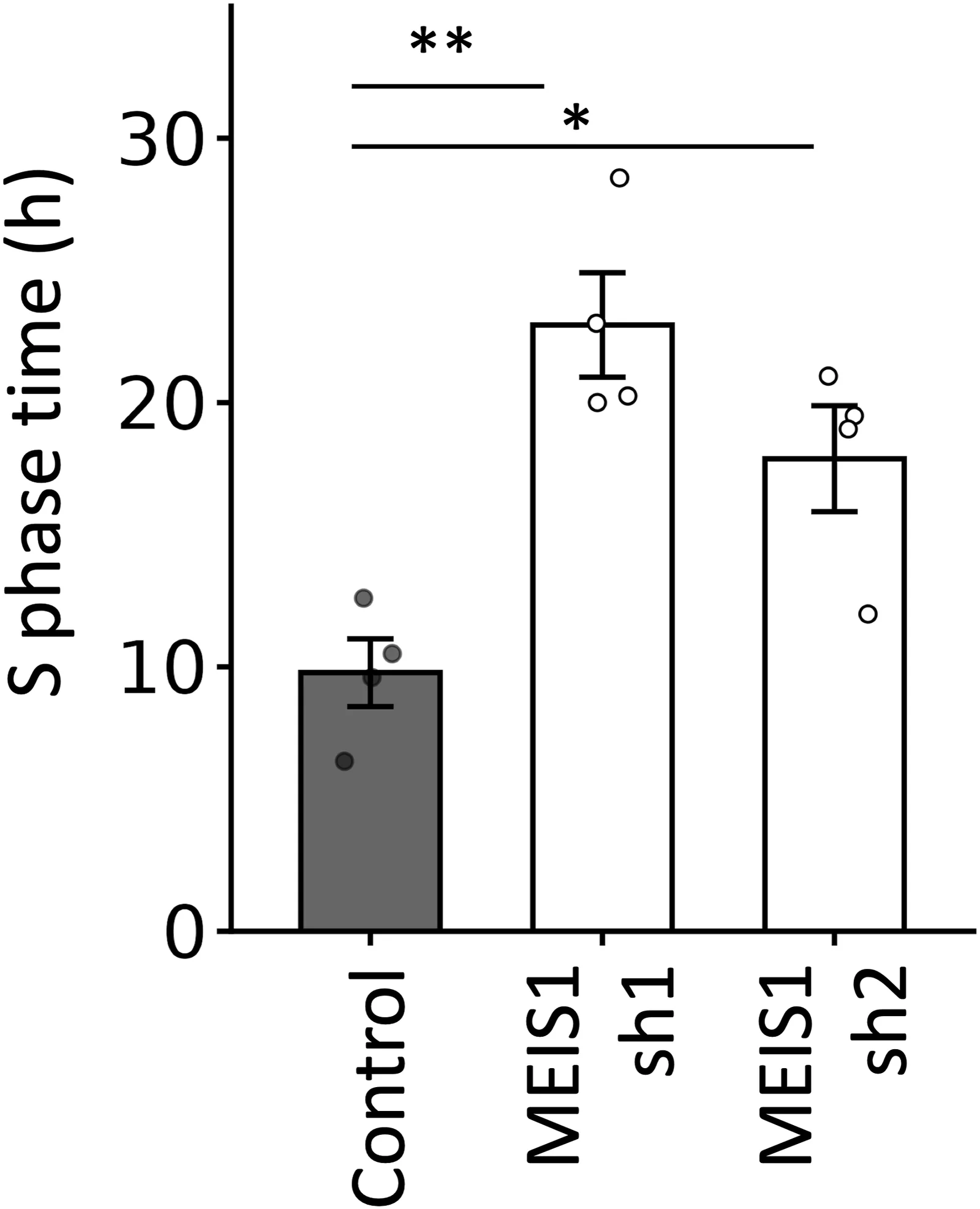
For S-phase duration quantification, sequential pulse-labeling with EdU and BrdU. Tukey’s test, *P < 0.05, **P < 0.01, n.s. = not significant. Values in the panels indicate mean ± SEM, n = 4.

However, in human brain organoids, *MEIS1* KD transiently increased the proportion of EdU+ proliferating cells. This observation likely reflected a mixed microenvironment containing both proliferative and differentiation signals. Therefore, to evaluate the effect of *MEIS1* depletion under a more controlled, proliferation-promoting condition, we performed *MEIS1* KD and EdU incorporation assays using cultured NPCs. *MEIS1* KD significantly reduced the proportion of EdU-positive cells by 66.3% in *MEIS1*-sh1 and 44.3% in *MEIS1*-sh2 (Fig 5C and D). Furthermore, to quantitatively determine the duration of the S-phase, we performed sequential EdU and BrdU pulse-labeling assays. This analysis revealed that *MEIS1* KD exhibited a distinct phenotype characterized by a prolonged S-phase duration. These findings suggest that *MEIS1* is essential for ensuring smooth and proper cell cycle progression in human NPCs (Fig S1). To further investigate the cellular basis of the growth defect, we examined whether the reduction in human NPC numbers after *MEIS1* KD was due to decreased proliferation or an increase in cell death. Immunostaining with an active Caspase 3 antibody was performed. However, there was no significant change observed in the proportion of active Caspase 3+ cells (Fig 5E and F).

### Human-biased *MEIS1-AS* KD down-regulated *MEIS1* mRNA expression

**Figure 6.**
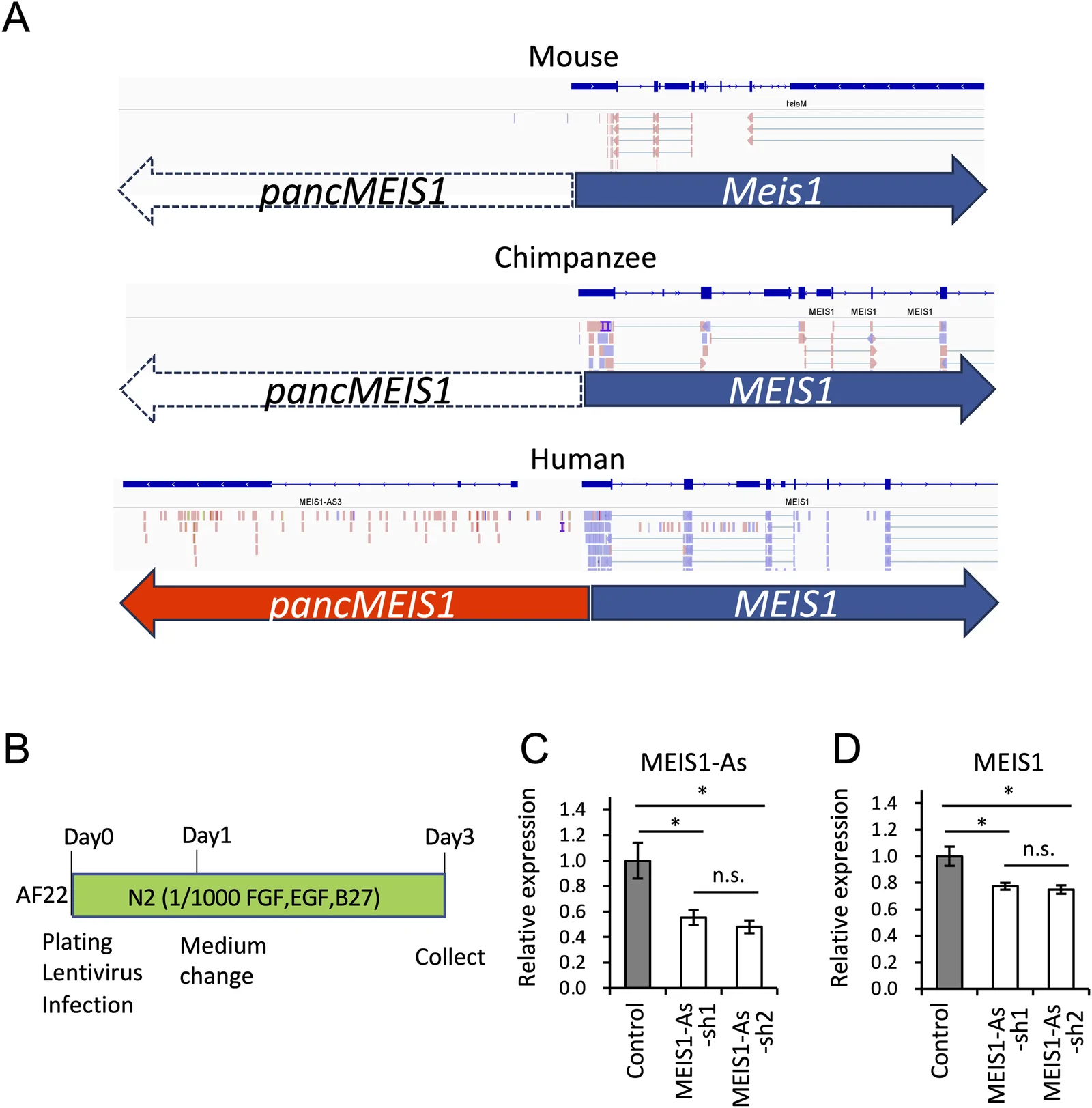
*MEIS1-AS* KD impaired MEIS1 expression. **(A)** Presence or absence of panc*MEIS1* in human and mouse. **(B)** Scheme for introducing shRNA into AF22 via lentivirus. **(C)** Quantification of *MEIS1*-AS KD Efficiency. **(D)** Quantification of *MEIS1* expression changes upon *MEIS1*-AS KD. Tukey’s test, *P < 0.05, n.s = not significant. Values in all panels indicate mean ± SEM.

**Figure S2.**
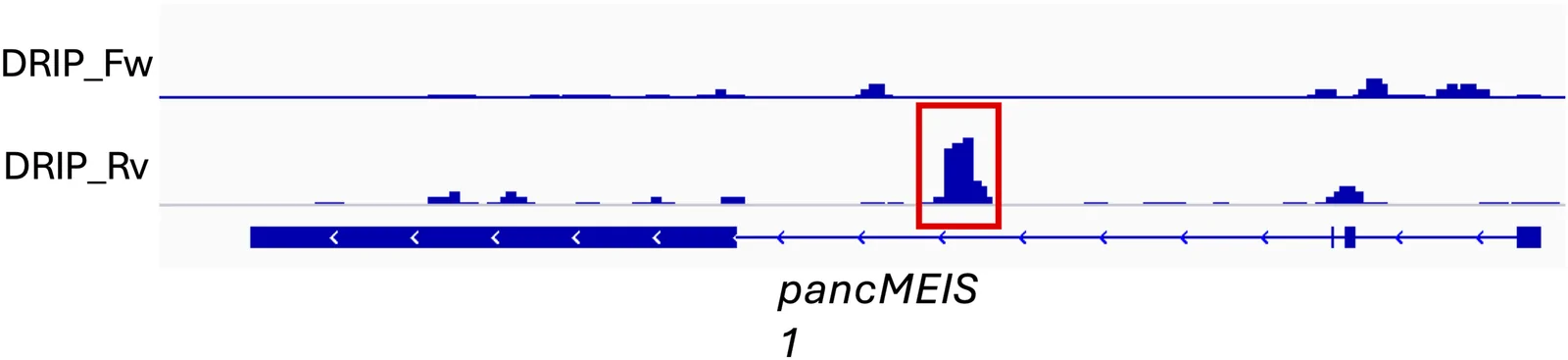
Visualization of DRIP-seq reads at the *pancMEIS1* locus using publicly available human NSC datasets. Signals corresponding to *MEIS1*-AS (panc*MEIS1*) is boxed.

ncRNAs have been known to be diverse among species. We focus on an ncRNA that has been registered only in humans as a reference transcript derived from *MEIS1* promoter (Fig 6A). When the public RNA-seq data of human NPCs, E15 mouse brain, and chimpanzee NPCs (Brattås et al, 2017; Sakai et al, 2018; Pal et al, 2025) were examined, this *MEIS1-AS* was actually expressed in humans, but not in mice and chimpanzee (Fig 6A). Analysis of publicly available DRIP-seq data in human NPCs (Yan et al, 2020) suggested that *pancMEIS1* forms an R-loop structure in a region ∼6 kb upstream of the *MEIS1* TSS (Fig S2). We investigated whether *MEIS1-AS* regulated *MEIS1* expression (Fig 6B). *MEIS1-AS* KD significantly reduced the expression of *MEIS1* (Fig 6C and D), which was in line with our previous studies showing that pancRNA activates the partner mRNA expression (Hamazaki et al, 2015). These results suggested that human-biased expression of *pancMEIS1* achieved high *MEIS1* expression in human NPCs.

### Human-biased *MEIS1-AS* affected NPC proliferation

**Figure 7.**
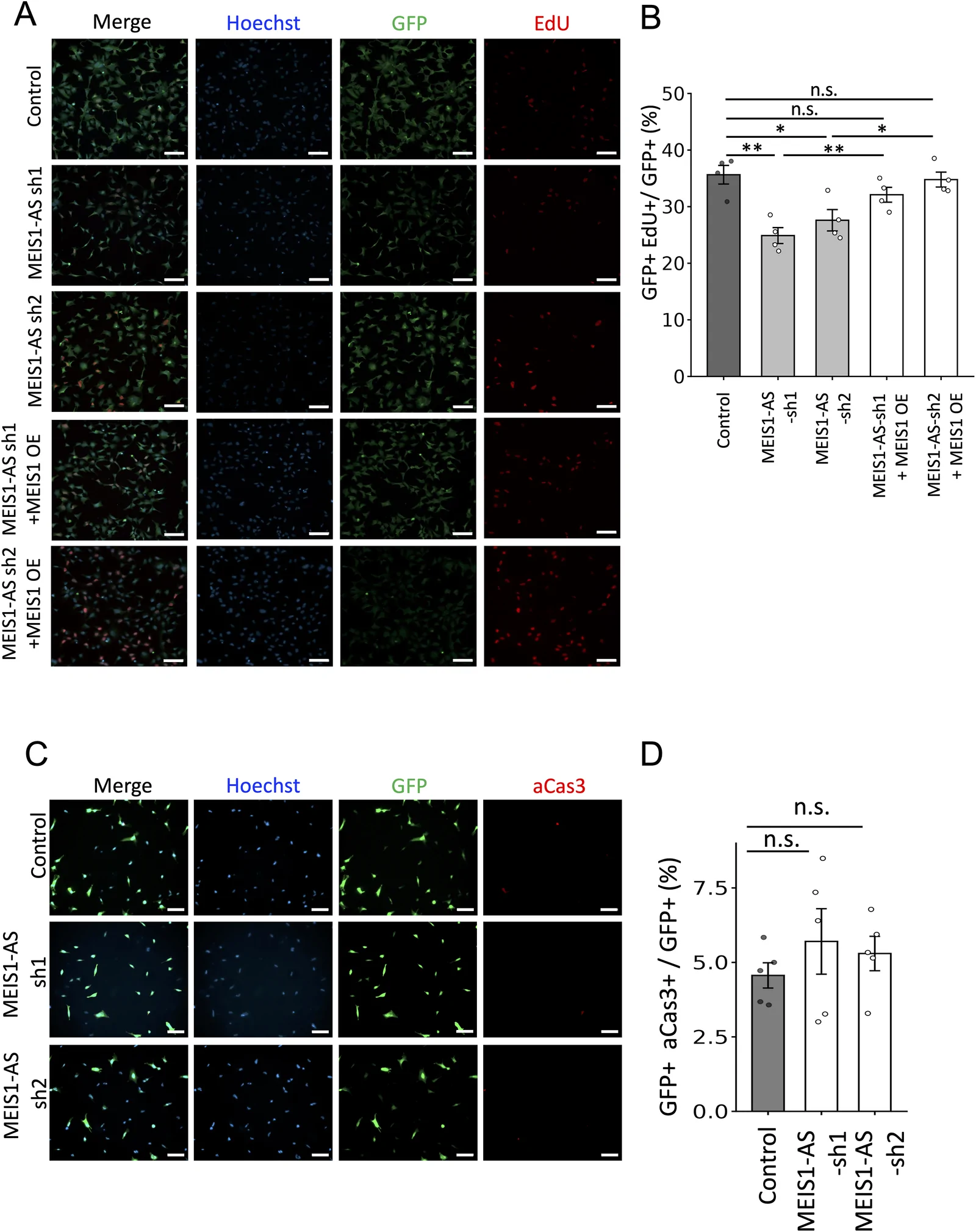
*MEIS1-AS* KD slowed cell cycle progression in iPSC-derived NPCs. **(A)** Representative images of EdU uptake experiment in AF22 stained for GFP (green) and EdU (pink) in control, *MEIS1*-AS KD, and *MEIS1*-AS KD + *MEIS1* OE. Nuclei were stained with Hoechst (blue). **(B)** Quantification of EdU+GFP+ to GFP+ cells in (A). **(C)** Representative images of immunocytochemistry stained for GFP (green) and active Caspase 3 (red) in control and *MEIS1*-AS KD. Nuclei were stained with Hoechst (blue). **(D)** Quantification of aCas3+GFP+ to GFP+ cells in (C). Tukey’s test, *P < 0.05, **P < 0.01, n.s. = not significant. Values in all panels indicate mean ± SEM, n = 5. Scale bar = 50 μm.

After *MEIS1-AS* KD, the cell proliferation rate was assessed through EdU incorporation experiments, and the proportion of cell death was evaluated by staining with an active Caspase 3 antibody. *MEIS1-AS* KD significantly reduced the proportion of EdU-positive cells by 34.2% in *MEIS1*-AS-sh1 and 42.9% in *MEIS1*-AS-sh2, which was effectively rescued by *MEIS1* overexpression (Fig 7A and B). However, there was no significant change observed in cell death (Fig 7C and D). These results were consistent with the EdU incorporation data obtained after *MEIS1* KD (Fig 5C and D). Therefore, human-biased pancRNA for *MEIS1* seemed involved in the proliferation of NPCs.

### *MEIS1* affected expression of genes related to cholesterol biosynthesis

**Figure 8.**
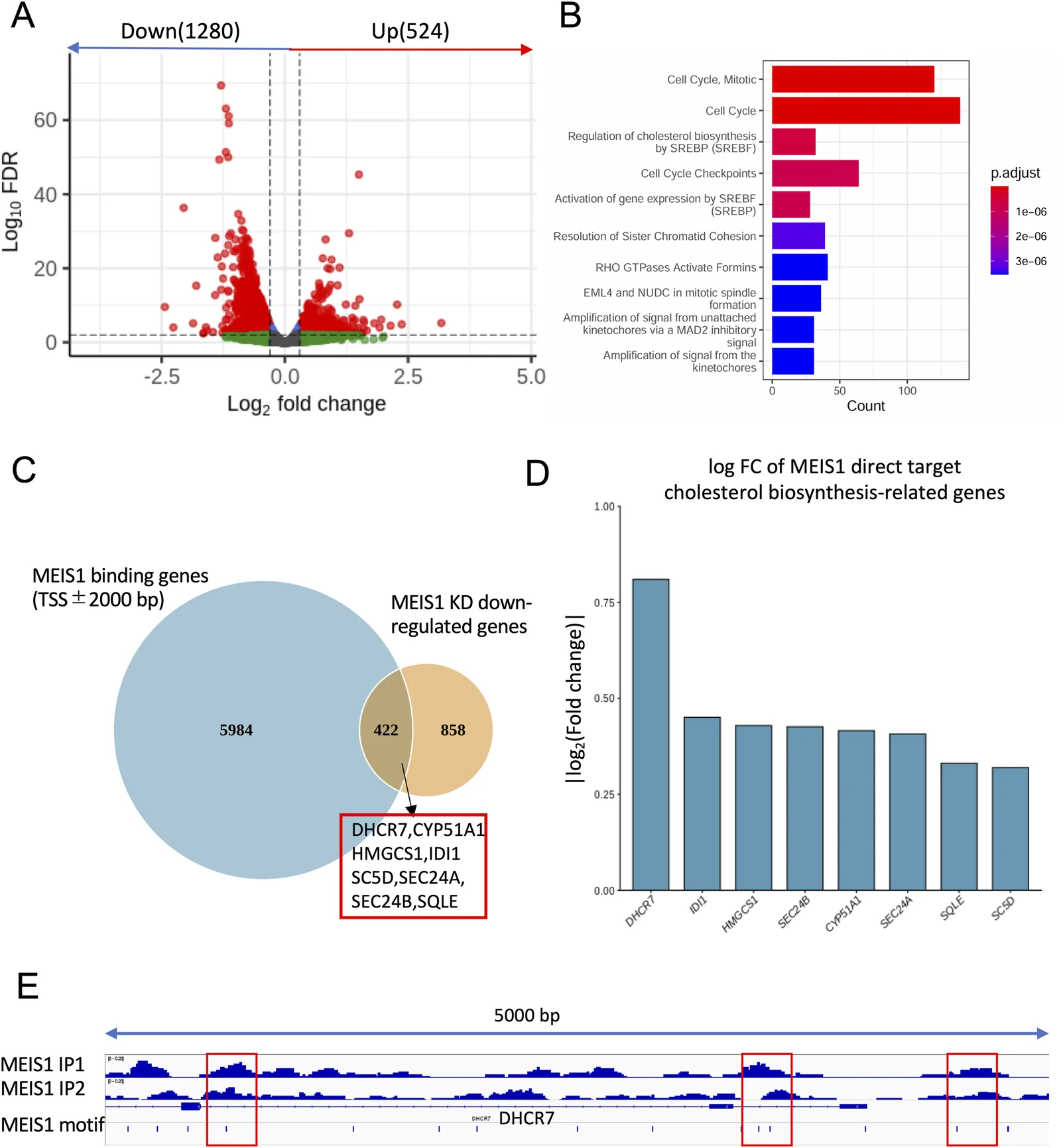
*MEIS1* regulated the expression of cholesterol biosysnthesis-related genes. **(A)** Volcano plot showing differentially expressed genes (DEGs) identified by RNA-seq in *MEIS1* KD human NPCs. **(B)** Pathway analysis of genes significantly down-regulated after *MEIS1* KD. **(C)** Venn diagram showing the overlap between ChIP-seq peaks and genes down-regulated upon *MEIS1* KD. **(D)** Expression changes of cholesterol biosynthesis–related genes that are direct targets of *MEIS1* and down-regulated upon *MEIS1* KD. **(E)** ChIP-seq signal visualization at the *DHCR7* promoter region.

To examine the transcriptome changes caused by *MEIS1* KD, RNA-seq analysis (n = 3) was performed (Fig 8A). |log_2_(fold change)| > 0.3 and q-value < 0.01 were defined as differentially expressed genes (DEGs), which were used for pathway analysis by Reactome database. For the down-regulated DEGs, mitotic cell cycle: R-HSA-69278, cell cycle: R-HSA-1640170, and regulation of cholesterol biosynthesis by SREBP (SREBF): R-HSA-1655829, activation of gene expression by SREBF (SREBP): R-HSA-2426168 were enriched (Fig 8B).

To identify the primary targets of *MEIS1*, we performed *MEIS1*-targeted ChIP-seq. A total of 5,984 peaks were identified. Among these, 422 peaks overlapped with down-regulated DEGs identified by RNA-seq upon *MEIS1* KD (Fig 8C). We further examined cholesterol biosynthesis-related genes within this set of 422 genes and narrowed them down to eight candidates (Fig 8C). Among these eight genes, *7-dehydrocholesterol reductase* (*DHCR7*) exhibited the largest expression change (Fig 8D). Three prominent peaks were identified in the vicinity of the *DHCR7* promoter (Fig 8E). In addition, all of these peaks showed greater than 90% similarity to the MEIS1-binding motif. Taken together with the RNA-seq data after *MEIS1* KD, these findings suggest that *MEIS1* recognizes the *DHCR7* locus in human NPCs.

### *DHCR7* overexpression rescued the proliferation defect caused by *MEIS1* KD

**Figure 9.**
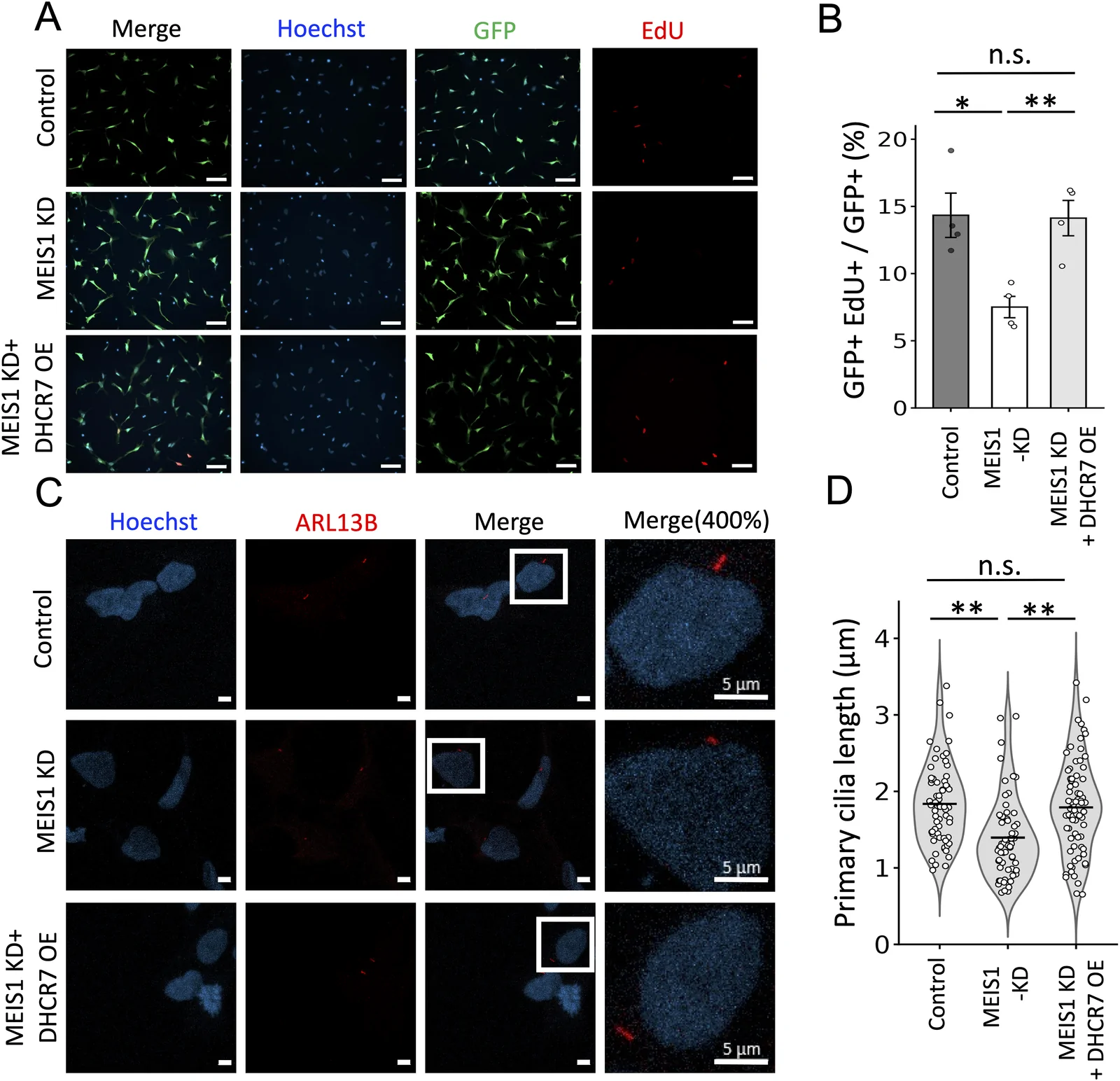
*DHCR7* overexpression rescued the proliferation defects caused by *MEIS1* KD. **(A)** Representative images of EdU uptake assays in AF22 cells expressing GFP (green) and EdU (magenta/pink) in control, *MEIS1* KD, and *MEIS1* KD + *DHCR7* OE groups. Nuclei were counterstained with Hoechst (blue). **(B)** Quantification of the ratio of EdU+GFP+ cells to total GFP+ cells. Tukey’s test, *P < 0.05, **P < 0.01, n.s. = not significant. Values in all panels indicate mean ± SEM, n = 4. Scale bar = 100 μm. **(C)** Representative images of primary cilia in AF22 cells, visualized by ARL13B staining (specific marker). Nuclei were counterstained with Hoechst (blue). **(D)** Quantitative analysis of primary cilia length. Tukey–Kramer test, *P < 0.05, **P < 0.01, n.s. = not significant. Values in all panels indicate mean ± SEM, n = 4, number of cilia (control: 65, *MEIS1* KD: 63, *MEIS1* KD + *DHCR7* OE: 75). Scale bar = 5 μm.

To investigate whether the defects caused by *MEIS1* KD could be rescued, we overexpressed *DHCR7*, a key enzyme in cholesterol biosynthesis, in *MEIS1*-KD human NPCs. Notably, *DHCR7* overexpression significantly restored the proportion of EdU-positive cells that had been decreased by *MEIS1* KD (Fig 9A and B). This result suggests that *DHCR7* as a critical downstream effector of *MEIS1* in regulating human NPC proliferation.

*DHCR7* is the causative gene for Smith-Lemli-Opitz syndrome (SLOS), a condition characterized by microcephaly and intellectual disability. Because SLOS is also categorized as a ciliopathy, we examined primary cilia morphology. We found that *MEIS1* KD shortened primary cilia length, and this phenotype was effectively rescued by *DHCR7* overexpression (Fig 9C and D).

## Discussion

We found that *MEIS1* expression is significantly higher in human APs and bRG compared with their mouse counterparts. Importantly, we identified human-biased expression of *pancMEIS1*, which positively regulates *MEIS1*. Mechanistically, we demonstrate that *MEIS1* is critical for maintaining metabolic programs required for organelle structure. Transcriptome analysis revealed that *MEIS1* depletion leads to the down-regulation of cholesterol biosynthesis genes. We identified *DHCR7* as a critical downstream effector; its overexpression rescued both the shortened primary cilia and the reduced proliferative capacity caused by *MEIS1* KD.

*MEIS1* functions as transcription factor that plays pivotal roles in pattern formation, cell proliferation, and cell fate determination during embryonic development. In the developing central nervous system, *MEIS1/Meis1* is known to regulate cerebellar granule cell progenitor proliferation, and mouse neocortical neurogenesis (Owa et al, 2018; Isogai et al, 2022).

Several previous studies have focused on human-specific genes as potential drivers to expand human neocortex (Florio et al, 2015; Liu et al, 2017). Because cortical folding has emerged progressively during evolution from fish to mammals and during mammalian evolution, multiple genes, not only specific genes, but also conserved genes, should be also involved in human BP expansion. In this study, we picked up *MEIS1* from human and mouse developmental brain datasets as a representative functionally important gene for brain development with human-biased expression in both aRG and bRG. *MEIS1* is more highly expressed in human neocortical NPCs compared with mice (Fig 1). Human-biased pancRNA was actually located nearby to be involved in the up-regulation of *MEIS1* (Fig 6). We will discuss the involvement of human-biased *MEIS1*/*Meis1* expression achieved by pancRNA in human-specific NPC behaviors to potentially differentiate the brain structures from those of other species.

Regarding NPC behaviors in a multicellular context, *MEIS1* KD accelerated the cell cycle of human NPCs followed by the increase in the number of differentiating neurons marked by DCX (Figs 2 and 3). It is not likely that the observed cell cycle progression expands NPC pools because 1 wk after the detection of cell cycle progression of NPCs, number of cells marked by either SOX2 was significantly decreased. Rather, cell cycle progression is a sign to enter into the premature differentiation stage that should exhaust the NPC pools, which can partly explain the smaller size of the mouse cortex due to the lower level expression of *Meis1* in mouse aRG and bRG. In fact, KD experiments showed that *MEIS1* positively regulated *PAX6* expression (Fig 5B), which was in line with a previous report using mouse samples (Owa et al, 2018). This suggests that the role of *MEIS1* in maintaining NPC stemness is conserved across species and that its high expression preserves the progenitor pool to ultimately generate more neurons. Whereas our present study establishes a primary role for *MEIS1* in driving the broad lineage transition from uncommitted neural stemness (SOX2) to early neuronal differentiation (DCX), its specific functions across distinct cell sub-types remain to be fully elucidated. Future studies using targeted lineage tracing in combination with subtype-specific markers—such as TBR2 for intermediate progenitor cells, HOPX for basal radial glia, and NGN1 for committed neurogenic progenitors—will be essential to clarify the cell type–specific responses triggered by human-biased *MEIS1* expression.

Forced expression of *MEIS1* in mice was performed by in utero electroporation to examine whether NPC proliferation could be enhanced in vivo. In the mouse context, BPs in the SVZ exhibit low proliferation because they are predominantly neurogenic basal intermediate progenitors (bIPs) with limited self-renewal capacity rather than highly proliferative bRGs. On the other hand, BP expansion supports the human neocortex expansion. Our results clearly showed that *MEIS1* up-regulation increased the proliferation of Pax6+ cells (Fig 4A and B). Contrary to the KD experiment of *MEIS1* in human cortical organoids where premature differentiation was induced, the proportion of Tbr2+ cells, a population in a transiently amplifying state after exiting the stem cell niche, was also reduced (Fig 3C and D). This suggests that observed cell cycle progression in our in utero electroporation humanization trial does not indicate premature differentiation, but rather it reflects an expansion of the NPC population, including within the SVZ. Consequently, evolutionary acquisition of pancRNA for *MEIS1* serves to maintain the NPC pools in both human aRG and bRG, as discussed further below.

At first glance, the contrasting EdU incorporation phenotypes observed upon *MEIS1* KD—a reduction in 2D cultured NPCs (AF22) versus a transient increase in human cortical organoids—might appear contradictory. However, we interpret these findings as reflecting distinct cellular contexts rather than an experimental discrepancy. In 2D monolayer cultures, where cells are maintained under homotypic, proliferation-saturated conditions, *MEIS1* depletion leads to a significant reduction in EdU incorporation, demonstrating that *MEIS1* plays a primary, cell-intrinsic role in promoting cell proliferation and sustaining cell cycle kinetics. Conversely, 3D cortical organoids represent a complex heterotypic niche where proliferative cues, cell–cell interactions, and fate-specification signals dynamically interplay. In such a multicellular architecture, cell cycle regulation is intimately coupled to neuronal differentiation and lineage progression (Lange et al, 2009). Thus, the transient EdU elevation after *MEIS1* KD in organoids does not contradict the role of *MEIS1* in promoting proliferation. Rather, as supported by the quantitative determination showing an extended S-phase duration upon *MEIS1* KD (Fig S1), we believe that *MEIS1* is fundamentally required for proper NPC proliferation.

The RNA-seq and ChIP-seq data suggested that *MEIS1* regulates the genes related to cholesterol biosynthesis (Fig 9B–D). All eight cholesterol biosynthesis-related genes that are direct targets of *MEIS1* and showed reduced expression upon *MEIS1* KD possessed ChIP-seq peaks overlapping MEIS1’s consensus sequence. This indicates that *MEIS1* mediates the expression regulation of multiple cholesterol biosynthesis-related genes. Importantly, overexpression of *DHCR7*, which showed the most pronounced expression change upon *MEIS1* KD, rescued the proliferation defect caused by *MEIS1* KD, strongly suggesting that *MEIS1* may regulate human NPC proliferation through cholesterol biosynthesis. *DHCR7* is an enzyme responsible for the final step in cholesterol synthesis, as evidenced by the fact that germline mutations in the *DHCR7* is associated with Smith-Lemli-Opitz syndrome (SLOS), an autosomal recessive disorder, multiple congenital malformation and intellectual disability syndrome, with clinical characteristics that encompass a wide spectrum and great variability (e.g., microcephaly, cleft palate, syndactyly of toes 2 and 3, polydactyly, visceral polydactyly, visceral malformations) (DeBarber et al, 2011). Particularly in the context of brain development, loss or deficiency of *DHCR7* has been shown to induce premature neurogenesis and deplete the cortical precursor pool in both murine and human neural progenitors (Tomita et al, 2022). This phenotype closely parallels our finding that *MEIS1* KD—and the accompanying down-regulation of *DHCR7*—drives human NPCs toward premature differentiation and pool exhaustion, further corroborating the critical role of the *MEIS1*–*DHCR7* axis in cortical progenitor maintenance. In this context, *DHCR7* may represent one of the key components of the *MEIS1*-cholesterol biosynthesis–related gene axis that regulates cholesterol levels in NPCs in a human-specific manner. This notion is supported by ChIP-seq data, which revealed three prominent peaks at the *DHCR7* promoter that overlapped with a known MEIS1-binding motif.

Fig 9B shows that *MEIS1* KD in human NPCs leads to shortening primary cilia, which is rescued by *DHCR7*. Consistent with this observation, cells from SLOS patients exhibit abnormal Sonic Hedgehog (Shh) signaling through cilia and that cholesterol supplementation restores Shh signaling. In addition, it is known that osteoblasts lacking *DHCR7* exhibit shortened primary cilia and reduced shh signaling (Suzuki et al, 2020), reinforcing the idea that *DHCR7* is physically linked for NPC maintenance through maintenance of cilia structure. Indeed, there are known cases in which changes in the properties of primary cilia in apical radial glia affect cell proliferation and differentiation (Ou et al, 2002; Snedeker et al, 2017; Shao et al, 2020; Cappuccio et al, 2022). Together, our findings suggest that human-biased *MEIS1* prolongs NPC stemness by modulating ciliary structure in radial glia via transcriptional control of *DHCR7*, a key enzyme in cholesterol biosynthesis. Although primary cilia assembly is tightly coupled to cell cycle exit, our finding that *DHCR7* overexpression rescues both ciliary length and NPC proliferation upon *MEIS1* depletion suggests a functional integration of ciliary integrity and proliferative capacity downstream of *MEIS1*. Nevertheless, to address alternative possibilities regarding the downstream mechanics of the *MEIS1*–*DHCR7* axis, it remains plausible that *DHCR7*-mediated cholesterol changes may also influence other cholesterol-rich membrane microdomains or cell signaling pathways independently of, or in parallel with, primary cilia structure. Given the complex reciprocal crosstalk between ciliary dynamics and cell cycle machinery, we cannot fully rule out these indirect feedback contributions or secondary structural adaptations, which warrant further investigation.

*MEIS1-AS* has been deposited as a reference noncoding RNA transcript, but its function remained unknown. Our previous studies have shown that “head-to-head” structure of RNA transcription has been frequently observed at CpG-rich sequences, named CpG islands (Uesaka et al, 2014). As such, pancRNAs move to the opposite direction to their partner genes and is functional in specifically up-regulating the partner genes (Hamazaki et al, 2015). Our data indicate that *MEIS1-AS* is a pancRNA for *MEIS1*, which has been evolutionary acquired to differentiate the human-biased expression of *MEIS1*, because the corresponding transcript does not exist in the mouse NPCs (Fig 6). Although our current functional characterization relied on human NPC models, exploring the effects of ectopic *MEIS1-AS* expression in vivo using embryonic mouse models represents an exciting avenue for future research to clarify how this human-biased lncRNA integrates into multicellular tissue architectures. Considering that much more species-specific pancRNAs compared with those for protein-coding genes have been discovered (Uesaka et al, 2017), it is interesting to speculate that finely tuning the expression pattern of conserved genes according to the species-specific context has been epigenetically accomplished, leading to the genome level acquisition of species-specific noncoding RNAs, if the epigenome adaptation to a given environment would be advantageous.

Our findings would have broad implications for understanding differences in brain development between species. In contrast to previous studies, this research demonstrates that species-specific differences in NPCs can arise from the differential regulation of genes with highly conserved sequences, driven by the presence of species-specific noncoding RNAs. Understanding species-specific brain evolution requires a focus not only on novel, species-specific genes, which have traditionally been the primary focus, but also on the divergent expression and regulation of highly conserved orthologous genes.

## Materials and Methods

### Animals

All aspects of animal care and treatment were performed according to the guidelines of the Experimental Animal Care Committee of Kyushu University (Fukuoka, Japan) and Hiroshima University (Higashi-Hiroshima, Japan). Timed-pregnant ICR mice (Japan SLC) were used in this study.

### Constructs

Lentivirus vectors used to express short-hairpin RNAs (shRNAs; pLLX), and FLAG-tagged MeCP2 (pLEMPRA) were provided by Dr. Z. Zhou (University of Pennsylvania School of Medicine) and Dr. M.E. Greenberg (Harvard Medical School). pLLX and pLEMPRA are dual-promoter lentivirus vectors constructed by inserting the U6 promoter-driven shRNA asette 5′ into the ubiquitin-C promoter in the FUIGW plasmid (Lois et al, 2002; Zhou et al, 2006). Empty vector was used as a negative control in KD and overexpression experiments. A FLAG-tagged human *MEIS1* expression lentivirus vector was constructed by replacing *MeCP2* with *MEIS1* at the EcoRI and AscI sites of pLEMPRA-MeCP2. A human *MEIS1* sequence was cloned by PCR using following primers:

Fw 5′-CGG​GAA​TTC​CAT​GGC​GCA​AAG​GTA​CGA​CGA-3′, Rv 5′-TTA​GGC​GCG​CCG​CTT​ACA​TGT​AGT​GCC​ACT​GCC-3′; shRNAs against *MEIS1* (sh1: GCG​TGG​CAT​CTT​TCC​CAA​AGT, sh2: GGG​ACT​CAC​CAT​CCT​TCA​AGT) and *MEIS1-AS* (sh1: GCT​GAA​CTG​AAA​GAG​GTT​GAG, sh2: GCT​GAA​ACC​TGG​AAG​ACA​CAA) were designed to target human genes.

### Cell culture

Human iPSC-derived NPCs (AF22) was used in this study. The culture dishes were coated with poly-L-ornithine (Sigma-Aldrich) and laminin (CORNING), and cells were seeded in hN2 medium (DMEM/F12, 2.5 μg/ml insulin, 100 μg/ml apo-transferrin, 16 μg/ml putrescine dihydrochloride, 30 μM sodium selenite, and 20 μM progesterone) supplemented with 1 μl/ml B27 (Thermo Fisher Scientific), 10 ng/ml bFGF (10018B; peproTech), 10 ng/ml EGF (AF10015; peproTech), and 10 μl/ml Antibiotic-Antimycotic Mixed Stock Solution (Nacalai Tesque). Cells were seeded at a density of 1.5 × 10^5^ cells per 35 mm culture dish and were cultured under conditions of 37°C and 5% CO_2_.

For lentivirus production, HEK293T was used. The culture medium consisted of DMEM high glucose (Nacalai Tesque) supplemented with 100 μl/ml fetal bovine serum (Biosera) and 10 μl/ml Antibiotic-Antimycotic Mixed Stock Solution (Nacalai Tesque). Cell passaging was performed 2 d after cell seeding.

### Lentivirus production

Lentiviruses were produced as described previously (Tsujimura et al, 2015). Briefly, lentivirus particles were constructed by cotransfecting HEK293T cells with the lentivirus constructs pCMV-VSV-G-RSV-Rev and pCAG-HIVgp using polyethyleneimine (Polysciences). The culture supernatants were collected 48 h after transfection, and virus was introduced into iPSCs, AF22 or cortical organoids by adding these supernatants to the culture media.

### Generation of cortical organoids

Cortical organoids were generated as described previously (Trujillo et al, 2019). Briefly, feeder-free iPSCs (provided by Dr. Hideyuki Okano), which were used as a control in a previous study (Nakashima et al, 2021), were cultured in mTeSR1 (ST-85850; StemCell Technologies) for 7 d. Colonies were dissociated using Accutase (Life Technologies) in PBS (1:1) for 10 min at 37°C and centrifuged for 3 min at 150*g*. The cell pellet was resuspended in mTeSR1 supplemented with 10 μM SB431542 (13031; Cayman Chemical) and 1 μM dorsomorphin (3,093; Tocris). Approximately 10,000 cells were transferred to one well of a 96-well plate (Corning 96-well clear round bottom ultra-low attachment microplate) and kept in suspension on an orbital shaker (95 rpm) in the presence of 5 μM ROCK inhibitor (Y-27632; Wako, 030-24021) for 24 h to form free-floating spheres. After 3 d, mTeSR1 was replaced with Media1 (Neurobasal supplemented with 20 μl/ml B27, 10 μl/ml GlutaMAX, 10 μl/ml N2 NeuroPlex [Gemini Bio-Products], 10 μl/ml NEAA [Nacalai Tesque], 10 μl/ml PS [Nacalai Tesque], 10 μM SB431542, and 1 μM dorsomorphin) for 7 d. Each organoid were embedded in 40 μl Matrigel (Corning) at day 9. The cells were then maintained in Media2 (Neurobasal with 20 μl/ml B27, 10 μl/ml GlutaMAX, 10 μl/ml NEAA, and 10 μl/ml PS) supplemented with 20 ng/ml FGF for 7 d, followed by 7 additional days in Media2 supplemented with 20 ng/ml FGF and 20 ng/ml EGF. Next, cells were transferred to Media3 (Media2 supplemented with 10 ng/ml BDNF [450-02; PeproTech], 10 ng/ml GDNF [450-10; PeproTech], 10 ng/ml NT-3 [450-03; PeproTech], 200 mM L-ascorbic acid [A92902; Sigma-Aldrich], and 1 μM dibutyryl-cAMP [11540-61; Nacalai Tesque]) to promote maturation, gliogenesis, and activity. After 7 d, cortical organoids were maintained in Media2 for as long as needed, with medium changes every 3–4 d. 10 μM Edu (Invitrogen Click-iT EdU Cell Proliferation Kit) was added to the culture media for 30 min. The organoids were fixed with 4% paraformaldehyde. The organoids were embedded with OCT compound (SAKURA), sliced at 20 μm with Cryostat (CM3050S; Leica) and used for immunohistochemistry. At least three rosettes per organoid were counted across multiple sections to obtain a single value for each organoid, and a minimum of three independent organoids (n ≧ 3) were analyzed per condition.

### In utero electroporation

In utero electroporation was performed as described previously (Nakashima et al, 2018). Briefly, electroporation was performed on E13.5 mouse embryos (male and female). Plasmid DNA (1.0 mg/ml in PBS containing 0.1% Fast Green) was injected into the lateral ventricle of the embryonic brain from outside the uterus. Fifty-millisecond electric pulses of 40 V were delivered five times at intervals of 950 ms using a model CUY21 Single Cell Electroporator (Nepa Gene). Glass micropipettes were prepared using a P-1000IVF (Sutter). Animals were perfused with 4% paraformaldehyde at E15.5 or E18.5. The brains were embedded with OCT compound (SAKURA), sliced at 20 μm with Cryostat (CM3050S; Leica) and used for immunohistochemistry. At least three sections per brain were counted to obtain a single value for each animal, and at least three brains from different litters were analyzed.

### Immunohistochemistry

Sections were washed with PBS, permeabilized and blocked with blocking buffer (3% FBS and 0.1% Triton X-100 in PBS) at room temperature (RT) and incubated with primary antibody diluted in blocking buffer at RT for 2 h or 4°C overnight. The following primary antibodies were used in this study: Goat anti-Sox2 (1:500; R&D Systems); chicken anti-GFP (1:1,000; Aves Laboratories); Rabbit anti-Pax6 (1:500; BioLegend); Rabbit anti-DCX (1:500; Abcam); Rabbit anti-Tbr2 (1:500; Abcam); Mouse anti-Phospho-Histone H3 (Ser10) (1:500; Cell Signaling); mouse anti-Satb2 (1:500; Abcam); rat anti-Ctip2 (1:500; Abcam); Rabbit anti-ARL13b (1:500; Proteintech); Mouse anti-BrdU (1:500; Proteintech). After cells were washed with PBS, they were incubated for 1 h at RT with the following corresponding secondary antibody: CF488 donkey anti-chicken IgG (HL), highly cross-adsorbed (1:500; Biotium); CF555 donkey anti-mouse IgG (HL), highly cross-adsorbed (1:500; Biotium); CF555 donkey anti-rabbit IgG (HL), highly cross-adsorbed (1:500; Biotium); CF647 donkey anti-goat IgG (HL), highly cross-adsorbed (1:500; Biotium) and CF647 donkey anti-rabbit IgG (HL), highly cross-adsorbed (1:500; Biotium). Hoechst 33258 (1:500; Nacalai Tesque) was used for nuclear staining. After a final wash with PBS, the cells were mounted on glass slides with Immu-Mount (Thermo Fisher Scientific). For EdU incorporation and Active Caspase 3 (aCas) assays, fluorescence images were acquired using a ZEISS Axio Observer equipped with an EC Plan-Neofluar 20×/0.5 Ph2 DIC II objective lens (NA 0.5, magnification: 20×). For organoid section analyses, fluorescence images were acquired using an FV3000 IX83 (Olympus) equipped with a UPLSAPO 20×/0.80 dry objective lens (NA: 0.80, magnification: 20x). For brain section analyses, fluorescence images were acquired using a confocal microscope (LSM800 or LSM900; Zeiss) equipped with a Plan-Apo 10x/0.45 Ph2 DIC II objective lens (NA 0.45, magnification: 10×). For primary cilia analyses, fluorescence images were acquired using a confocal microscope (LSM800 or LSM900; Zeiss) equipped with a Plan-Apo 100×/1.4 Oil DIC II objective lens (NA 100, magnification:1.4×).

For the quantitative analysis of primary cilia length, immunofluorescence images were acquired as Z-stacks. To measure the cilia length accurately in three-dimensional space, maximum intensity projections (MIP) were generated from the Z-stack images using ImageJ/Fiji software. Cilia were identified by ARL13B immunostaining, and their length was manually traced and measured using the S Straight Line tool in ImageJ. For each condition, more than 20 individual primary cilia were quantified per dish, across three independent dishes (n = 3 biological replicates).

### BrdU/EdU double staining

For BrdU/EdU double staining, cells were incubated with 10 μM BrdU (Cat# 08779-61; Nacalai Tesque) for 30 min and then cultured in fresh medium without BrdU for 3 h. 10 μM EdU was subsequently added for 30 min before fixation with 4% paraformaldehyde. EdU incorporation was detected using the Click-iT EdU Cell Proliferation Kit for Imaging (Invitrogen) with Alexa Fluor 555 or Alexa Fluor 647 according to the manufacturer’s instructions. Briefly, fixed cells were incubated with the Click-iT reaction cocktail for 40 min at room temperature. After washing with PBS, samples were treated with DNase I (20 U/ml) at 37°C for 30 min to allow antibody access to incorporated BrdU. Cells were then permeabilized and blocked with blocking buffer (3% FBS, 0.1% Triton X-100 in PBS). Primary antibodies diluted in blocking buffer were then applied for 2 h at room temperature. After washing with PBS, cells were incubated with the corresponding secondary antibodies for 1 h at room temperature. Hoechst 33258 (1:500; Nacalai Tesque) was used for nuclear staining. After a final wash with PBS, samples were mounted with Immu-Mount (Thermo Fisher Scientific), and then, images were acquired using a ZEISS Axio Observer 7 (ZEISS).

According to the dual-thymidine analog–labeling principle (Nowakowski et al, 1989; Martynoga et al, 2005), S-phase length (Ts) was calculated using a BrdU/EdU leaving-fraction assay. Cells that remained EdU^+^ at the time of fixation (BrdU^+^EdU^+^) were considered to have remained in the S-phase throughout the 3 h chase, whereas BrdU^+^EdU^−^ cells were considered to have exited the S-phase during this interval. Ts was calculated as Ts = Ti × Scells/Lcells, where Ti is the interval between the BrdU and EdU pulses (3 h), Scells is the number of EdU^+^ cells (cells in the S-phase at fixation), and Lcells is the number of BrdU^+^EdU^−^ cells (cells that had left the S-phase).

### EdU assay

Cells were incubated with EdU using the Click-iT EdU Cell Proliferation Kit for Imaging (Invitrogen) with Alexa Fluor 555 or Alexa Fluor 647 dye for 30 min. After incubation, cells were fixed with 4% paraformaldehyde and then stained with the Click-iT reaction buffer at room temperature for 40 min.

### qRT-PCR analysis

Total RNAs were isolated with Sepasol-RNA I Super G (Nacalai Tesque) and subjected to reverse transcription with the SuperScript VILO cDNA Synthesis Kit (Invitrogen) according to the manufacturer’s instructions. qRT-PCR was performed using a KAPA SYBR Fast qPCR Kit (Kapa Biosystems) with ROX as the reference dye (Thermo Fisher Scientific) with the StepOne Real-Time PCR System (Applied Biosystems). Expression levels of each gene were normalized to GAPDH and calculated relative to the control. The following primers were used:

GAPDH:

Fw: 5′-ACC​ACA​GTC​CAT​GCC​ATC​AC-3′

Rv: 5′-TCC​ACC​ACC​CTG​TTG​CTG​TA-3′

*MEIS1*:

Fw: 5′-TCT​GCA​CTC​GCA​TCA​GTA​CC-3′

Rv: 5′-TGG​CGA​ACA​CGG​CTA​TAT​CT-3′

PAX6:

Fw: 5′-CCG​GCA​GAA​GAT​TGT​AGA​GC-3′

Rv:5′-TTTCCCAAGCAAAGATGGAC-3′

SOX2:

Fw: 5′-GCT​ACA​GCA​TGA​TGC​AGG​ACC​A-3′

Rv: 5′-TCT​GCG​AGC​TGG​TCA​TGG​AGT​T-3′

*MEIS1*-AS:

Fw: 5′-CTG​GCT​TCT​CCC​TCC​TTT​TC-3′

Rv: 5′-CAG​CGG​GAG​TTA​TTT​TGC​AC-3′

### DRIP-seq data analysis

Publicly available strand-specific DRIP-seq datasets for hNSCs (SRR11185387 and SRR11185388) were obtained from a previous study (Yan et al, 2020). Adapter and quality trimming were performed using Trim Galore v0.6.6. Trimmed reads were aligned to the GRCh38/hg38 reference genome using Bowtie2, and alignments were coordinate-sorted using Samtools (Li et al, 2009). PCR duplicates were removed using MarkDuplicates (Picard Tools). For strand-specific analyses, strand-specific BAM files (forward/Watson and reverse/Crick) were separated using Samtools. Strand-specific coverage tracks were generated using deepTools bamCoverage (Ramírez et al, 2014) with CPM normalization (--normalizeUsing CPM) and a 50-bp bin size (--binSize 50), and exported as BigWig files.

### RNA-seq and data analysis

For publicly available RNA-seq analysis, reads were processed with the FASTX tool kit (Patel & Jain, 2012) to remove short (< 20 bp) and low-quality (quality score < 20) reads, followed by trimming of the adapter sequence. Processed reads were aligned to genome assembly mm10 or hg38 using TopHat (Kim et al, 2013). To compare gene expression changes, we used TPM values.

For the analysis of publicly available RNA-seq datasets, raw reads were processed using the FASTX-Toolkit to remove short (<20 bp) and low-quality (quality score < 20) reads, followed by adapter sequence trimming. The processed reads were aligned to the mouse (mm10) or human (hg38) reference genomes using TopHat (Trapnell et al, 2009). Gene expression levels were quantified as transcripts per million (TPM) for comparative analyses.

For RNA-seq library preparation and sequencing of AF22 cells, total RNA was extracted from AF22 cells using Sepasol-RNA I Super G (Nacalai Tesque) and the NucleoSpin RNA kit (Takara). Poly(A) mRNA was isolated using the NEBNext Poly(A) mRNA Magnetic Isolation Module (New England Biolabs) according to the manufacturer’s instructions. Sequencing libraries were constructed using the NEBNext Ultra II Directional RNA Library Prep Kit for Illumina (New England Biolabs). cDNA libraries were size-selected with AMPure XP beads (Beckman Coulter) and their quality was assessed using an Agilent 2100 Bioanalyzer. Sequencing was performed on the DNBSEQ-G400 platform (BGI) with 150-bp paired-end reads.

Adapter sequences and low-quality bases were trimmed using Trim Galore. The resulting reads were aligned to the human reference genome (hg38) using STAR (Dobin et al, 2013). Gene-level read counts were generated using BEDTools (Quinlan & Hall, 2010), and expression levels were quantified as TPM for downstream comparative analyses.

### ChIP-seq and data analysis

Briefly, AF22 cells were harvested and crosslinked with fixation solution for 10 min at room temperature, followed by quenching with glycine. Cells were lysed in SDS lysis buffer supplemented with RNase and protease inhibitors and incubated on ice. Chromatin was fragmented by sonication, and insoluble debris was removed by centrifugation.

Antibody-conjugated beads were prepared in advance by blocking with 0.5% BSA in PBS. Immunoprecipitation was performed using 4 μl (1:125 dilution of anti-*MEIS1* antibody [ab19867; Abcam]) per IP reaction in a total volume of 500 μl containing chromatin derived from 3 × 10^6^ cells. The supernatant was diluted with ChIP dilution buffer and incubated with precleared beads, followed by overnight immunoprecipitation with antibody-conjugated beads at 4°C.

After immunoprecipitation, beads were sequentially washed with RIPA buffer containing 150 mM NaCl, RIPA buffer containing 500 mM NaCl, Buffer III, and TE buffer. Bound complexes were eluted and treated with Proteinase K, followed by reverse cross-linking. RNA was further treated with DNase I and purified by acid phenol–chloroform extraction and ethanol precipitation. Purified DNA was resuspended in TE buffer.

Library preparation was performed using the NEBNext Ultra II DNA Library Prep Kit for Illumina (E7645; NEB) according to the manufacturer’s instructions.

Adapter sequences and low-quality bases were trimmed using Trim Galore. The resulting reads were aligned to the human reference genome (hg38) using Bowtie2 (Langmead & Salzberg, 2012). BigWig files were generated using bamCoverage with CPM normalization, and peak calling was performed using MACS2 (Zhang et al, 2008).

### Quantification and statistical analysis

No statistical methods were used to pre-determine sample sizes. No data points were excluded. We do not use any methods to determine whether the data met assumptions of the statistical approach. All data were collected and processed randomly. Unpaired *t* tests were used to calculate the *P*-value for pairwise comparisons. For multiple comparisons, *P* values were calculated using Tukey’s multiple comparison’s test. The exact values of n (sample size) are provided in the figures and figure legends. We considered a *P*-value less than 0.05 to be statistically significant. Data represent mean + SEM.

## Supplementary Material

Reviewer comments

## Data Availability

The raw sequencing data have been deposited in Gene Expression Omnibus under accession number GSE322827 (RNA-seq) and GSE323332 (ChIP-seq).
