# Supplementary material for: Human-biased MEIS1 expression by a promoter noncoding RNA regulates neural stem cell proliferation
Source: Life Sci Alliance. 2026 Sep 30;9(12):e202603708. doi: 10.26508/lsa.202603708 (PMC13627767; doi:10.26508/lsa.202603708)
Supplement: Supplementary file 1 [file LSA-2026-03708_review_history.pdf]

Human-biased MEIS1 expression by a promoter non-coding RNA regulates neural stem cell proliferation

Hayato Uchida, Tomonori Kameda, Shota Zenno, Hideyuki Nakashima, Yuki Minato, Yuina Aritsuka, Sota Nishida, An Boyang, Mizuki Honda, Fumihiro Morishita, Kinichi Nakashima, and Takuya Imamura
DOI: https://doi.org/10.26508/lsa.202603708

Corresponding author(s): Takuya Imamura, Hiroshima University

Review Timeline:

Submission Date:	2026-03-21
Editorial Decision:	2026-04-30
Revision Received:	2026-08-04
Editorial Decision:	2026-09-09
Revision Received:	2026-09-14
Accepted:	2026-09-18

Scientific Editor: Sarita Hebbar

Transaction Report:

(Note: With the exception of the correction of typographical or spelling errors that could be a source of ambiguity, letters and reports are not edited. The original formatting of letters and referee reports may not be reflected in this compilation.)

April 30, 2026

Re: Life Science Alliance manuscript #LSA-2026-03708-T

Prof. Takuya Imamura
Hiroshima University
Graduate School of Integrated Sciences for Life
Kagamiyama 1-3-1
Higashi-Hiroshima, 7398526
Japan

Dear Dr. Imamura,

Thank you for submitting your manuscript entitled "Human-specific gene-activating non-coding RNA targets MEIS1 for cortical expansion" to Life Science Alliance.
Your manuscript was reviewed by three expert reviewers whose comments are appended below. As you will read, the reviewers have all acknowledged the potential value of this work but have also raised several significant concerns. In line with their overall assessment, we invite you to submit a revised manuscript addressing the reviewers' comments.

We agree with the reviewers that following overstated or unsubstantiated claims must be supported by some evidence.
-description of bRG-like characteristics upon MEIS1 expression in mouse cortex (Reviewer 1, point 2 and Reviewer 3, point 1).
-the broader evolutionary conclusion and the human-specific nature of MEIS1-A, please provide comparative analyses as suggested by the reviewers or at least moderate the claim (Reviewer 1, point 1, Reviewer 2, point 13, and Reviewer 3, point 7).
-accelerated cell cycle transition (all reviewers). Please provide the necessary evidence as requested by the reviewers or temper this claim.

We highlight the reviewers common concerns on incomplete descriptions, and contradictory results with MEIS1 knockdown in organoids versus NPCs
-We concur with the reviewers that you must explain this contradiction (Reviewer 1, point 4, Reviewer 2, point 8, Reviewer 3, point 3).
-We also agree that descriptions must be more in-depth and accurate (eg. include other markers, NPC sub-types; Reviewer 2, points 4-6, Reviewer 3, points 4-6).

-In agreement with Reviewers 1 and 3, we ask you to include the possibility that MEIS1-AS knockdown effects might reflect MEIS1-independent functions of the non-coding RNA whilst discussing the data, or provide rescue data.

-We agree that the cholesterol measurements are not necessary for consideration at LSA. However, you must address the relevant points raised by the reviewers whilst discussing the connection to cholesterol biosynthesis, cilia morphology, and neocortical expansion (Reviewer 1, point 6, Reviewer 2, points 10-12).

When submitting the revision, please include a letter addressing the reviewers' comments point by point. While a rebuttal must respond to all points in some form, additional experiments to resolve these points, other than indicated above, will not be required.

I would be happy to discuss the revision in more detail via email or phone/videoconferencing. Please let me know which option you prefer, if any.

To upload the revised version of your manuscript, please log in to your account: https://lsa.msubmit.net/cgi-bin/main.plex

You will be guided to complete the submission of your revised manuscript and to fill in all necessary information. Please get in touch in case you do not know or remember your login name.

The typical timeframe for revisions is three months. Please note that papers are generally considered through only one revision cycle, so strong support from the referees on the revised version is needed for acceptance.

While you are revising your manuscript, please also attend to the below editorial points to help expedite the publication of your manuscript. Please direct any editorial questions to the journal office.

Thank you for this interesting contribution to Life Science Alliance. We hope that the comments below will prove constructive as your work progresses, and we are looking forward to receiving your revised manuscript.

Sincerely,

Sarita Hebbar, PhD
Scientific Editor
Life Science Alliance
http://www.lsajournal.org

A. THESE ITEMS ARE REQUIRED FOR REVISIONS

-- A letter addressing the reviewers' comments point by point.

-- An editable version of the final text (.DOC or .DOCX) is needed for copyediting (no PDFs).

-- High-resolution figure, supplementary figure and video files uploaded as individual files: See our detailed guidelines for preparing your production-ready images, https://www.life-science-alliance.org/authors

-- Summary blurb (enter in submission system): A short text summarizing in a single sentence the study (max. 200 characters including spaces). This text is used in conjunction with the titles of papers, hence should be informative and complementary to the title and running title. It should describe the context and significance of the findings for a general readership; it should be written in the present tense and refer to the work in the third person. Author names should not be mentioned.

-- By submitting a revision, you attest that you are aware of our payment policies found here: https://www.life-science-alliance.org/copyright-license-fee

B. MANUSCRIPT ORGANIZATION AND FORMATTING:

Full guidelines are available on our Instructions for Authors page, https://www.life-science-alliance.org/authors

We encourage our authors to provide original source data, particularly uncropped/-processed electrophoretic blots and spreadsheets for the main figures of the manuscript. If you would like to add source data, we would welcome one PDF/Excel-file per figure for this information. These files will be linked as supplementary "Source Data" files.

IMPORTANT: It is Life Science Alliance policy that if requested, original data images must be made available. Failure to provide original images upon request will result in unavoidable delays in publication. Please ensure that you have access to all original microscopy and blot data images before submitting your revision.

Reviewer #1 (Comments to the Authors (Required)):

This is an interesting and potentially important manuscript. The central idea is conceptually strong and rather original. The study combines a variety of approaches and model systems which is appreciated. At the same time, the manuscript currently overstates several of its conclusions, as detailed below.

Major concerns:
1. The manuscript presents MEIS1-AS as a human-specific regulatory mechanism contributing to cortical expansion, but the comparative evidence is essentially limited to human versus mouse analyses. This is insufficient to support a broader evolutionary conclusion. The claims should be narrowed, or additional comparative analysis should be provided. My suggestion is to rephrase this claim to "a human-associated regulatory transcript absent in mouse and functionally relevant in human NPCs".
2. MEIS1 overexpression in embryonic mouse cortex increases PAX6+ and PH3+ cells and reduces Tbr2+ cells in SVZ/IZ of 3 embryos. This may be interpreted as consistent with altered progenitor state, but these data do not demonstrate bRG identity or "humanization" which is mentioned. Further analyses are needed to support the bRG identity. Morphological analysis (using GFP for example) is required as well as better marker-based evidence (combination of markers PAX6 and TBR2, addition of phospho-vimentin) to support the interpretation.
3. Further and related to the previous, 2-day acute overexpression is not enough to claim that MEIS1 drives cortical expansion. For this, it would be necessary to extend incubation time to perinatal stages, show increased cortical thickness, folding or increased neuronal numbers in the mice and possibly a similar effect on organoid size or growth.
4. In cortical organoids, MEIS1 KD increases EdU acutely in SOX2+ cells, then later reduces SOX2 and increases DCX, which the authors interpret as accelerated cell-cycle transition followed by premature differentiation and exhaustion. On the opposite, in AF22 NPCs, MEIS1 KD instead decreases the fraction of EdU-positive cells. These outcomes may be biologically reconcilable, but the manuscript does not really resolve the discrepancy. At minimum, the paper needs a clearer temporal model and ideally

direct cell-cycle measurements. Right now the explanation remains incomplete.
5. MEIS1-AS knockdown lowers MEIS1 mRNA and reduces EdU incorporation, which is consistent with the proposed model, but it does not yet prove that MEIS1-AS acts as a cis-activating ncRNA at the MEIS1 locus. The key missing pieces are rescue and locus-level mechanism. The authors should ideally show that re-expression of MEIS1 rescues the MEIS1-AS knockdown phenotype, and they should better distinguish whether the effect is mediated by the transcript itself.
6. The RNA-seq/ChIP-seq part and the DHCR7 rescue are interesting, given that overexpression of DHCR7 rescues proliferation and cilia-length phenotypes. It would be good to show that cholesterol levels are truly impacted upon MEIS1 kd/DHCR7 oe and whether this impacts cell morphology. Lastly, Since MEIS1 perturbation changes proliferation/differentiation, cilia shortening could also be a secondary effect rather than a direct mechanism.
7. In general, the manuscript would also benefit from clearer reporting of biological replication and choice of statistical analysis, particularly for organoid, in utero electroporation, and cilia measurements. The sample numerosity is typically very small which requires claims to be more conservative.

Minor concerns:
1. In figure 1, panel A is missing a y axis label, so it's not clear what quantity is being shown (raw or normalized counts?).
2. Incomplete sentence in results ("To identify the primary targets of MEIS1, we performed MEIS1-targeted ChIP-seq using ...")
3. Cross-species MEIS1 expression analysis could be improved by adding other datasets and extending the comparison to other species beyond mouse.

Reviewer #2 (Comments to the Authors (Required)):

This manuscript by Uchida et al aimed to demonstrate the role of the human-specific gene-activating non-coding RNA that targets MEIS1 in cortical development and evolutionary expansion. The authors explored their research questions across multiple model systems, including mice, organoids, and cultured progenitor cells, using various methodologies and techniques. Findings from this study will certainly advance the field of research. The manuscript's flow is logical and smooth, but additional information is needed to make the story cohesive. The data presented are compelling; however, most figures and results sections require deeper, more thorough analysis to support the manuscript's main claim. The sample size throughout the manuscript remains very low (n=3), which compromises the overall quality of the data. The following are detailed comments and concerns I have.

1. Although the authors mentioned their previous work sought to understand how human-specific pancRNAs mediate the formation of human-specific mRNA transcriptomes to alter NPC behaviours, why MEIS1 is the gene of interest and the focus of the study is not properly explained or justified. There should be more information in the introduction on this, as it establishes the foundation of the whole study and the rationale of the research.

2. The results section started with comparing the MEIS1 expression between humans and mice, using previously published transcriptomic data. The methods used for this comparison were not described; how did the authors account for sequencing or study variation between the two datasets? Figure 1A is missing a title and unit on the y-axis; there were no asterisks labelled in the figure panel, but the legend indicated $P<0.01$. This needs to be explained.

3. Other cell types need to be labelled in Figure 2B, especially those that have high expression of MEIS1 as well. The authors also indicated that this was analyzed using the Wang et al. dataset, but there is an inconsistency regarding whether single-cell or single-nucleus RNA-seq was used between the results text and the figure legend.

4. The results section reporting Figures 2 and 3 needs a more suitable title. "Accelerated the transition from the cell cycle towards neuronal differentiation" is very confusing; it sounds as though the MEIS1 KD changes cell-cycle length. Further quantification of the intermediate progenitor population is missing. Other markers, including TBR2, NGN1, and HOPX, should be used to support the claim here that MEIS1 pushes NPCs down the differentiative lineage. Analysis should also be done in separate cortical zones, VZ and SVZ.

5. Comparable regions in the organoids should be selected for analysis in Figure 3. The cortical thickness in the control group is clearly much thinner than in the KD groups, which could significantly bias the data and analysis. The binning or proportion of GFP+ cells across different cortical zones should be reported alongside marker quantification. Are results reported one week (indicated in text) or 10 days (indicated in Figure) after lentiviral infection?

6. The quality of Figure 4 is very low. There are arbitrary white lines that I assume indicate the border between VZ and SVZ; these lines are misaligned, and some extend beyond the image. A larger sample size is needed in general, but particularly for Figure 4B. PH3 is a mitotic marker, not a proliferation marker. Neuron abundance should be analyzed at a later time point, such as E17.5.

7. The endogenous level of MEIS protein in cortical organoids and embryonic mouse brain should be assessed to understand

the physiological expression of MEIS1 during development.

8. It is not clear why the authors decided to use cultured NPCs as a model to repeat the MEIS KD experiment, especially since the phenotype in the cortical organoid is rather clear. What advantage does NPC culture bring compared to organoids? This should be well justified. Moreover, data from MEIS KD in NPCs seems to contradict data from MEIS KD in organoids regarding EdU-positive cells (Figure 5C,D vs. Figure 2B,C). This should also be explained and discussed.

9. The format and style of Figure 8 are different from all other figures. Why the authors suddenly use (A) Left; Right to label different panels, rather than consistently using individual panel labels for images and quantification, is puzzling... Please be consistent.

10. More data are needed to connect MEIS1, DHCR7, cholesterol synthesis and cilia morphology. The current writing in the results and discussion sounds very chunky and random. With the sequencing date, the connection between MEIS1 and DHCR7 is justified. The authors should perform additional experiments focusing on the functions of MEIS1 and DHCR7, and on cholesterol synthesis. Why one particular disorder-related phenotype - cilia length was chosen to study needs more justification as well. If the authors aim to report that DHCR7, cholesterol synthesis, and cilia morphology are mechanisms by which MEIS1 contributes to brain development, they should be examined in all three model systems and in the two species used in this study.

11. The current approach to evaluating cilia length is not appropriate. Static images can only capture the stained organelle from one particular angle. Without 3D reconstruction or EM imaging, quantification of ciliary morphology and length is prone to large errors.

12. The discussion demands depth. The following should be discussed: MEIS1 function in the developing brain; MEIS1 regulates cholesterol biosynthesis in a broader biological context; Cholesterol synthesis and metabolism in the developing brain, species difference? Previous findings on DHCR7, especially in the developing brain?

13. Overall, the findings in the present study are more on the fundamental side, with less focus on the evolutionary expansion of the human brain. I would suggest that the authors choose a different title for the manuscript that better summarizes the role of MEIS1 in NPC biology, rather than the role of human-specific ncRNA in cortical expansion, especially since the study does not include any cross-species manipulative experiment addressing cortical expansion.

Minor points:
One sentence can not be a paragraph.
"... in vivo mouse brain" should be "... in mouse brain in vivo"
Typos including: TBR22,
Figures 5E, F belong to the supplement.
Figures 6A, B belong to the supplement.
Figure 7 legend (A) needs to be corrected.
Page 8, second paragraph, sentence 1 is incomplete.

Reviewer #3 (Comments to the Authors (Required)):

In the manuscript "Human-specific gene-activating non-coding RNA targets MEIS1 for cortical expansion" by Uchida et al., the authors investigate the role of MEIS1 and its neighboring human-specific non-coding RNA pancMEIS1 in human brain development. Using a combination of knockdown and overexpression approaches in brain organoids, embryonic mouse neocortex, and in vitro differentiated neural progenitor cells (NPCs), the authors provide evidence that pancMEIS1 might regulate MEIS1 expression. Furthermore, they suggest that MEIS1 likely influences the proliferative capacity and differentiation potential of NPCs. These are interesting and potentially important findings that could justify publication in Life Science Alliance. However, in its current form, the manuscript requires substantial revision before it can be considered for publication.

Major Comments:
1. The authors state in the Introduction that "mimicking human-type MEIS1 expression in mouse cortices induced bRG-like characteristics." However, throughout the manuscript, bRGs are neither quantified nor characterized. This claim should either be supported with appropriate data (e.g., marker analysis such as HOPX, morphological criteria) or removed.
2. The authors do not provide validation of MEIS1 knockdown efficiency. Evidence such as qRT-PCR, Western blot, or immunofluorescence should be included to demonstrate that MEIS1 expression is indeed reduced.
3. In MEIS1 knockdown brain organoids, the proportion of EdU-positive NPCs is significantly increased. This appears inconsistent with other findings: MEIS1 overexpression in embryonic mouse increases proliferation (Fig. 4C), whereas MEIS1 knockdown in in vitro NPCs decreases EdU incorporation (Fig. 5D). The authors should reconcile these seemingly contradictory results.
4. The conclusion that "MEIS1 KD accelerated the cell cycle transition of human NPCs" is not sufficiently supported. The authors did not measure cell cycle length. Additionally, Which NPC subtypes are affected (aRG, bIP, bRG)? Why is SOX2 staining absent in basal regions outside the VZ? What evidence supports accelerated cell cycle progression?

5. In Figures 4E and 4F, a decrease in Tbr2-positive progenitors is reported. What is the effect on neuronal populations? Are neuron numbers also reduced?
6. The authors conclude that high MEIS1 expression maintains NPC stem cell properties, suggesting a human-specific feature. However, which NPC subtype is affected? Tbr2+ cells decrease while PH3+ mitotic cells increase in the VZ. Does this indicate bRG-like cells? Additional marker analysis (e.g., HOPX) would be important to support this interpretation and strengthen the central claim.
7. The designation of pancMEIS1 as "human-specific" is insufficiently supported. While it may be absent in mouse, analysis across other primates is required before making this claim.
8. MEIS1-AS knockdown reduces MEIS1 expression only modestly (~20%). This raises the question of whether the observed phenotypes are mediated via MEIS1 or reflect MEIS1-independent functions of the non-coding RNA.
9. It would be informative to test whether ectopic expression of MEIS1-AS in embryonic mouse increases MEIS1 expression.
10. For RNA-seq analysis, the authors use a relatively lenient cutoff (log2 fold change > 0.3). More stringent thresholds may help identify more robust and biologically relevant candidates.

Minor Comments:
1. Figure 1A lacks units. Please specify whether the data represent TPM, relative expression, or arbitrary units.
2. The dataset from Fietz et al. (2012) includes multiple fetal stages. Please indicate which developmental stage(s) are shown.
3. There is a discrepancy in Figure 2: the text states that organoids were derived from MEIS1 KD iPSCs, whereas the schematic suggests lentiviral knockdown in organoids. Please clarify.
4. In Figure 3, the authors mention changes in Pax6+ cells, but Pax6 data are not shown.
5. Phospho-histone H3 is described as a proliferation marker; more precisely, it is a mitotic marker.
6. Figure 5A is not referenced in the text.
7. The sentence "To identify the primary targets of MEIS1, we performed MEIS1-targeted ChIP-seq using" is incomplete.
8. The statement "In mouse context, BPs in SVZ usually have low proliferation because largely they are not bRGs any more and have begun to differentiate into neurons" is inaccurate. In mice, basal progenitors are primarily bIPs and have not yet differentiated into neurons.

Reply to Reviewer #1

Major concerns:

1. Human-specificity of *MEIS1-AS*

Reviewer comment: The manuscript presents *MEIS1-AS* as a human-specific regulatory mechanism contributing to cortical expansion, but the comparative evidence is essentially limited to human versus mouse analyses. This is insufficient to support a broader evolutionary conclusion. The claims should be narrowed, or additional comparative analysis should be provided. My suggestion is to rephrase this claim to "a human-associated regulatory transcript absent in mouse and functionally relevant in human NPCs".

Response: We sincerely thank the reviewer for this constructive and insightful comment. We agree that restricting comparative evidence to human versus mouse is insufficient to firmly establish a broader evolutionary conclusion regarding human-specific traits.

To address this limitation and thoroughly test the evolutionary conservation of *pancMEIS1*, we newly analyzed RNA-seq data derived from chimpanzee NPCs. As shown in the newly added Integrative Genomics Viewer (IGV) snapshot shown below, *pancMEIS1* expression is clearly detected in human NPCs, whereas it is completely absent in both mouse and chimpanzee NSCs.

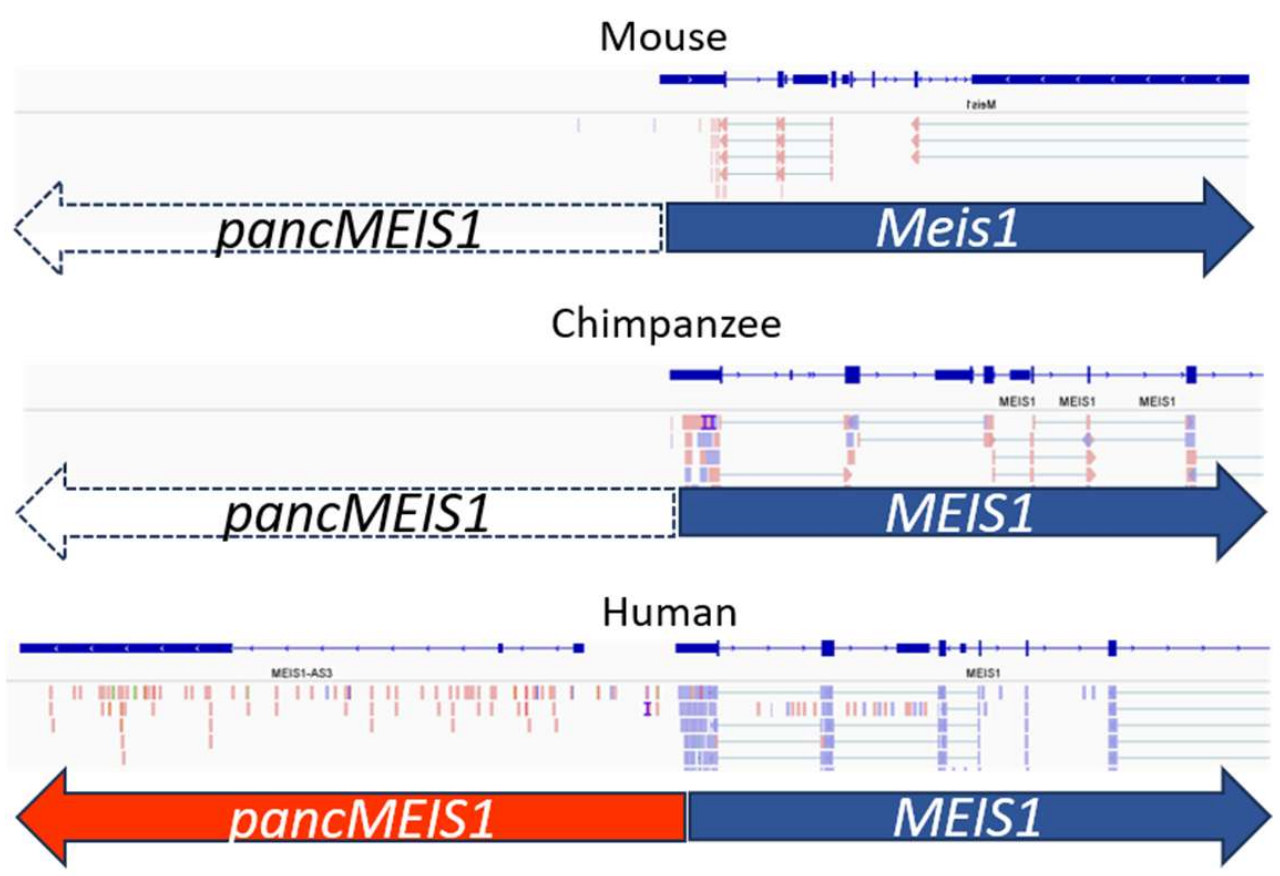

Accordingly, we have updated the text to include the relevant information.
page 7, lines 184–187, Fig. 6A

2. bRG identity / "humanization" claim

Reviewer comment: *MEIS1* overexpression in embryonic mouse cortex increases PAX6+ and PH3+

cells and reduces Tbr2+ cells in SVZ/IZ of 3 embryos. This may be interpreted as consistent with altered progenitor state, but these data do not demonstrate bRG identity or "humanization" which is mentioned. Further analyses are needed to support the bRG identity. Morphological analysis (using GFP for example) is required as well as better marker-based evidence (combination of markers PAX6 and TBR2, addition of phospho-vimentin) to support the interpretation.

Response: We agree with the reviewer that Pax6+/PH3+ positivity and decreased Tbr2+ proportion alone are insufficient to definitively establish basal radial glia (bRG) identity without cell morphology analysis or specific bRG markers such as phospho-vimentin.
Rather than claiming a specific induction of bRG identity, we have softened our statements to focus on the broader effect of *MEIS1* on NPC proliferation. Specifically, we have removed direct references to bRG identification in the mouse cortex and revised the manuscript to describe these cells as proliferating mouse NPCs and SVZ progenitors at the last part of the Introduction section (brief summary of the coming Results section). The revised text emphasizes that *MEIS1* expression promotes the expansion and maintenance of the overall NPC pool without explicitly claiming the acquisition of bRG-specific cellular features.

Accordingly, we have updated the text to include the relevant information.
Page 4, line 102-104

3. Duration of overexpression / "cortical expansion" claim

Reviewer comment: Further and related to the previous, 2-day acute overexpression is not enough to claim that *MEIS1* drives cortical expansion. For this, it would be necessary to extend incubation time to perinatal stages, show increased cortical thickness, folding or increased neuronal numbers in the mice and possibly a similar effect on organoid size or growth.

Response: We appreciate this important point. According to the reviewer's suggestion, we performed additional experiments to observe the prolonged effects of *MEIS1* overexpression in mice. We found that *MEIS1* overexpression by in utero electroporation at E18 significantly increased the number of both CTIP2-positive cells (deep-layer neurons marker) and SATB2-positive cells (upper-layer neurons marker). These findings suggest that *MEIS1* contributes to increase the neuronal populations in each layer.

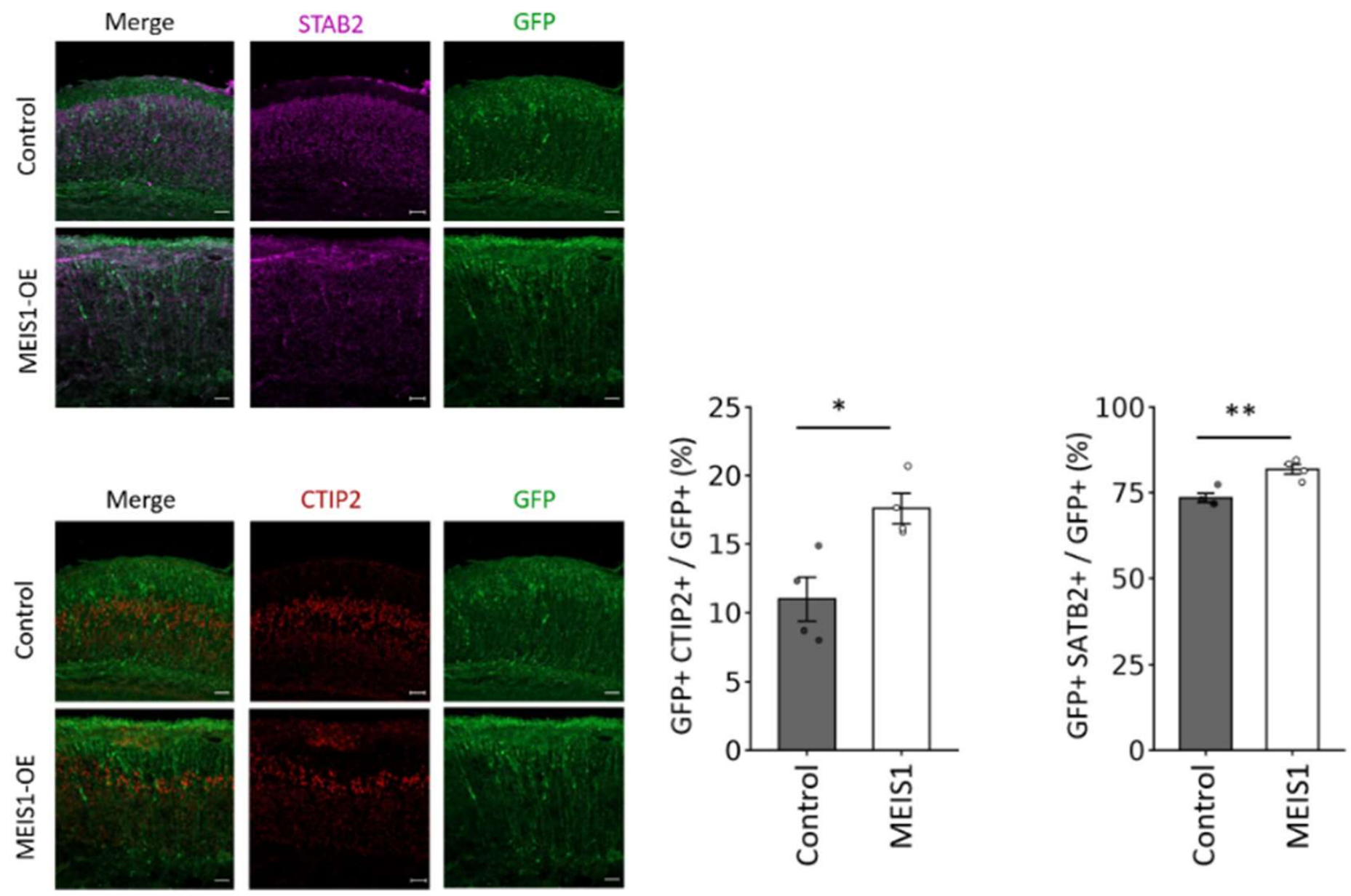

Accordingly, we have updated the text to include the relevant information.

page 6, lines 150–153, Fig. 4G-J

4. Discrepancy between organoid and 2D NPC (AF22) results

Reviewer comment: *MEIS1* KD increases EdU/SOX2 then later decreases SOX2/increases DCX in organoids (accelerated cycling → premature differentiation), but decreases EdU in AF22 NPCs. This discrepancy needs a clearer temporal model and direct cell-cycle data to reconcile.

Response: We sincerely thank the reviewer for this insightful comment. We agree that a clearer temporal model and direct cell-cycle analysis are essential to fully reconcile the observed phenotypes between 2D NPCs (AF22) and 3D cortical organoids. To directly address this point, we have newly quantified the S-phase duration in AF22 NPCs. We found that MEIS1 knockdown significantly extended the S-phase duration (Fig. S1). This new result directly demonstrates that *MEIS1* plays a crucial role in proper cell-cycle kinetics. Regarding the seemingly contradictory phenotypes between 2D monolayer cultures and 3D cortical organoids, we reason that these outcomes reflect fundamental differences in their cellular environments. In 2D monolayer cultures (AF22): Under pure, homotypic, and proliferation-saturated conditions, the extended S Phase and impaired cell-cycle kinetics lead to a straightforward reduction in the overall fraction of EdU-positive cells. In 3D cortical organoids: In a complex multicellular niche, cell-cycle progression is intimately coupled with fate-specification signals. In this context, the acute S-phase delay/stalling

caused by *MEIS1* depletion may trigger an accelerated cell-cycle exit, which initially manifests as a transient accumulation of EdU-labeled cells, followed by premature differentiation and ultimate depletion of the progenitor pool.

Accordingly, we have updated the text to include the relevant information.
page 7, lines 170–174, Fig. S1
page 12, lines 291–305

5. Mechanism of *MEIS1-AS* as *cis*-acting ncRNA

Reviewer comment: Knockdown data are consistent with the model but don't prove direct cis activation at the *MEIS1* locus. Need rescue experiment (*MEIS1* re-expression) and locus-level mechanistic evidence.

Response: We appreciate this important point. We additionally performed rescue experiments by overexpressing *MEIS1* in *pancMEIS1 KD* cells. MEIS1 overexpression significantly rescued the proliferation defect induced by pancMEIS1 depletion, demonstrating that *pancMEIS1* regulates cell proliferation in a MEIS1-dependent manner. Moreover, analysis using public strand-specific DRIP-seq identified an R-loop peak at the *MEIS1* promoter locus (SRR11185387 and SRR11185388: Yan *et al.*, Cell Rep. 32, 107870, 2020), raising the possibility that *pancMEIS1* functions in *cis* at the *MEIS1* promoter to promote *MEIS1* expression through an R-loop-dependent regulatory mechanism.

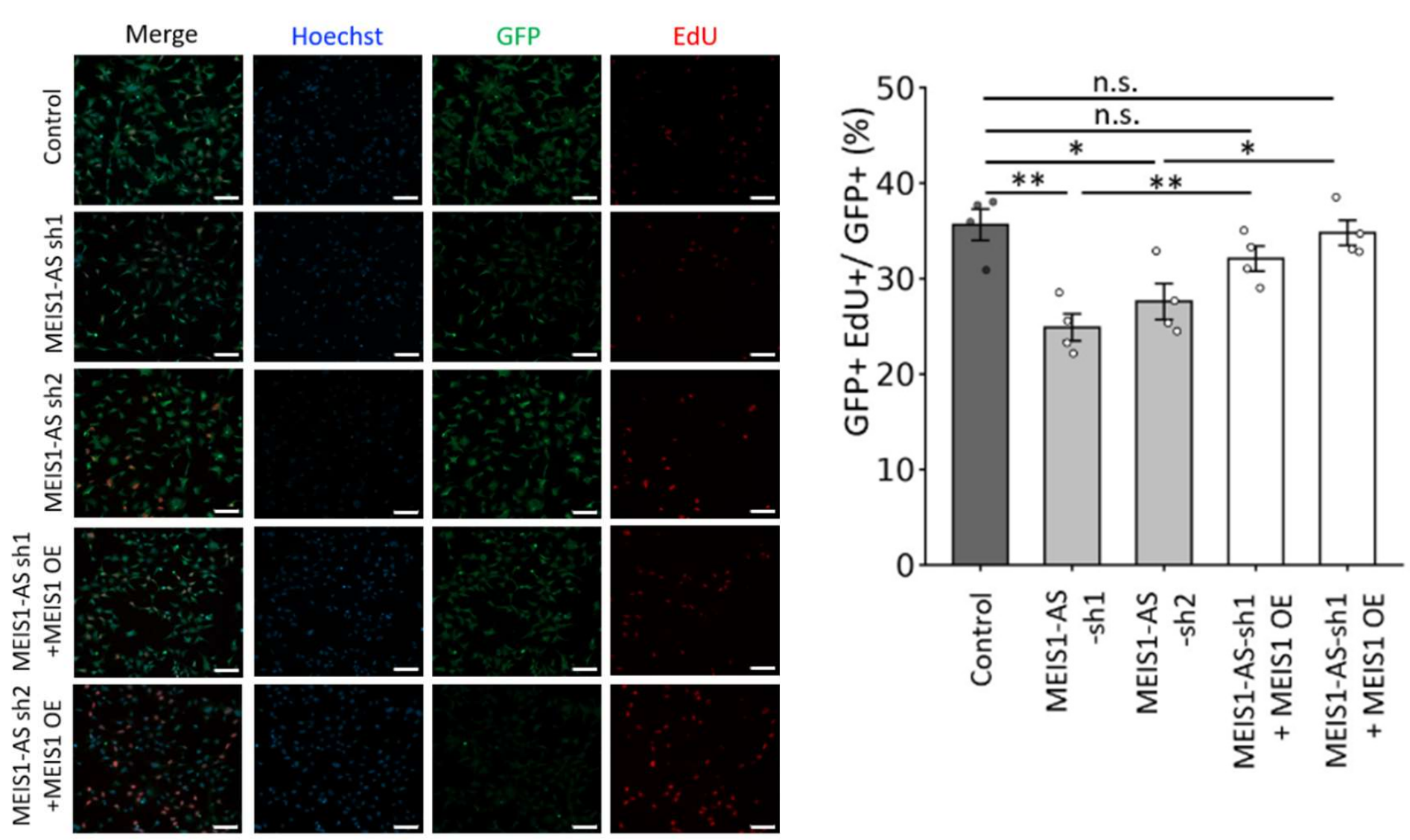

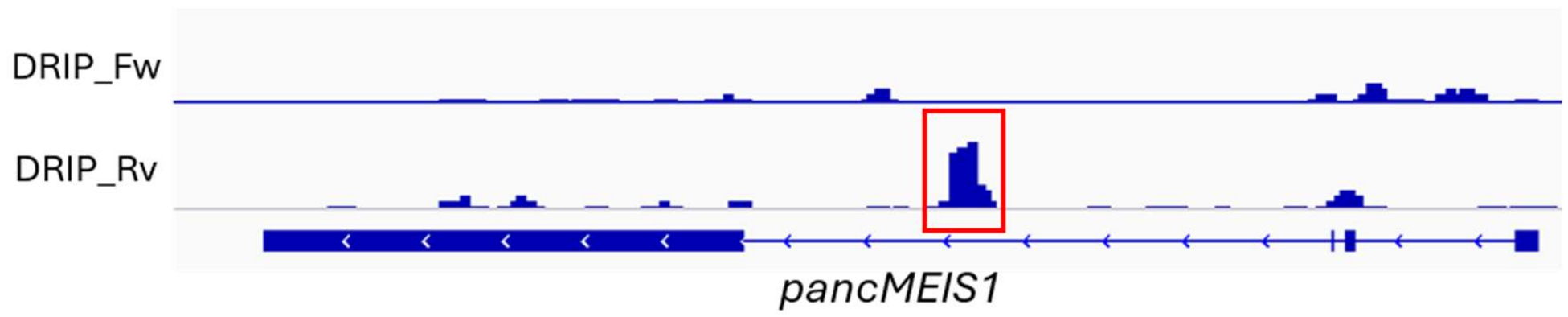

Accordingly, we have updated the text to include the relevant information.
page 8, lines 196–201, Figs. 6E and F
page 8, lines 187-189, Fig. S2

6. cilia secondary effect

Reviewer comment: cilia shortening could also be a secondary effect rather than a direct mechanism.

Response; We thank the reviewer for these insightful comments regarding the downstream mechanisms of the MEIS1–DHCR7 axis.

Regarding cholesterol measurement and cell morphology, *DHCR7* is well-established as the key terminal enzyme in the Kandutsch-Russell pathway of cholesterol biosynthesis. Extensive literature has shown that alterations in *DHCR7* expression directly dictate intracellular cholesterol levels, which in turn affect cholesterol-rich membrane microdomains such as the primary cilium(Suzuki et al., 2020).

Nevertheless, to address the reviewer's concern rigorously, we have updated the Discussion section to include the alternative possibility that the reviewer has raised.
page 14, lines 340–352

Clarification of statistical methods, biological replicates, and adoption of conservative claims

Reviewer comment: Reporting of biological replicates and statistical methods needs improvement; small sample sizes call for more conservative claims.

Response: We appreciate the reviewer's helpful feedback regarding the reporting of statistical analyses and biological replication.

To ensure full transparency and clarity, we have thoroughly revised the manuscript as follows:

Materials and Methods: We have updated the Sentence regarding the biological replicates (page 19, lines 452-454; page19, lines 465-467; page 20, lines 488-494).

Fig. Legends: We have comprehensively reviewed all figure legends to explicitly state the exact

sample sizes (n), the precise definition of error bars (e.g., mean±SEM), the statistical tests applied for each panel.

Minor Comments:

1. y axis label in Fig. 1 panel A

Reviewer comment: In Fig. 1, panel A is missing a y axis label, so it's not clear what quantity is being shown (raw or normalized counts?).

Response: We have updated the y-axis label in Fig. 1A to explicitly specify TPM.

2. Incomplete sentence in results

Reviewer comment: Incomplete sentence in results ("To identify the primary targets of MEIS1, we performed MEIS1-targeted ChIP-seq using ...")

Response: We thank the reviewer for bringing this grammatical oversight to our attention. We have corrected the sentence on page 9, line 215 to clearly state the methodology:
"To identify the primary targets of MEIS1, we performed MEIS1-targeted ChIP-seq."

Cross-species comparison

Reviewer comment: Cross-species *MEIS1* expression analysis could be improved by adding other datasets and extending the comparison to other species beyond mouse.

Response: To further validate the human-specificity of *pancMEIS1* (*MEIS1-AS*), we extended our RNA-seq analysis to include chimpanzee neural progenitor cells (NPCs) alongside human and mouse datasets.

Remarkably, this comparative analysis revealed that *pancMEIS1* is exclusively expressed in human NPCs, whereas it is completely absent in both mouse and chimpanzee counterparts (Page 7, line 184-187, Fig. 6A). This finding directly demonstrates that *pancMEIS1* is not a general primate trait, but rather a strictly human-specific non-coding RNA that was evolutionary acquired to drive the human-biased upregulation of *MEIS1*.

Reply to Reviewer #2

1. Rationale for choosing MEIS1

Reviewer comment: The reason *MEIS1* was selected as the gene of interest is not adequately justified in the introduction.

Response: We sincerely thank the reviewer for pointing out the need for a clearer rationale behind selecting *MEIS1* as the main focus of our study.
In response to this comment, we have expanded the Introduction section to explicitly describe why MEIS1 was prioritized as a novel candidate target. Specifically, *MEIS1* is well known for driving neural progenitor regulation during cerebellar foliation— tissue folding structurally analogous to cerebral cortical gyrification (Owa et al., 2018). Furthermore, previous studies utilizing in utero electroporation in mouse embryonic cortices demonstrated that forced expression of Meis1 promotes neural progenitor proliferation (Isogai et al., 2022). Building upon these findings, our revised Introduction now clearly frames our central hypothesis: how the evolutionary emergence of a human-specific pancRNA at the *MEIS1* locus (*pancMEIS1*) tuned *MEIS1* expression to contribute to human neocortical expansion.

Accordingly, we have updated the text to include the relevant information.
page 4, lines 91–99

2. Human-mouse comparison methodology / Fig. 1A

Reviewer comment: Methods for cross-dataset comparison (batch/study variation) aren't described. Fig. 1A lacks a title, y-axis units, and significance asterisks despite the legend citing $P<0.01$.

Response: We thank the reviewer for bringing these important details to our attention.
Regarding the comparative transcriptomic analysis between human and mouse: The human and mouse RNA-seq datasets used for this comparison were derived from the same published study (Florio et al., 2015, Fietz et al., 2012) and were processed using identical library preparation protocols and sequencing platforms. Because the data originated from a unified study rather than disparate external sources, batch effects and inter-study sequencing variations were inherently minimized. Regarding the lack of several information on Fig.1A, we have updated this point.

3. Fig. 2B cell-type labeling and dataset inconsistency

Reviewer comment: Other *MEIS1* high expression cell types should be labeled in Fig. 2B. Text and figure legend disagree on whether single-cell or single-nucleus RNA-seq (Wang et al. dataset) was used.

Response: We thank the reviewer for pointing out these important details regarding Fig 2B and the methodology.
- Cell Type Labeling in Fig. 2B: As suggested, we have updated Fig. 2B to include explicit cell-type labels for all distinct cell clusters identified on the UMAP plot.

- We apologize for the inconsistency in the manuscript. We have corrected both the main text and the figure legends (including the legend for Fig. 2) to explicitly and consistently state that the dataset from Wang et al. was generated using single-nucleus RNA-sequencing (snRNA-seq).

4. Figs 2-3 title and marker analysis

Reviewer comment: Title is misleading (implies changed cell-cycle length); need intermediate progenitor quantification, more markers (TBR2, NGN1, HOPX), and separate VZ/SVZ analysis.

Response: We thank the reviewer for these valuable comments and suggestions regarding the presentation of our findings in Figs 2 and 3.

- We agree that the original figure title was confusing and could be misinterpreted as implying a change in cell-cycle kinetics/length. To state our conclusion more accurately, we have revised the title to: "Accelerated neuronal differentiation upon *MEIS1* knockdown"

- In our experimental design, we observed a transient increase in EdU incorporation as early as 3 days post-infection with sh-*MEIS1* lentivirus. To determine whether this brief proliferative burst reflects a physiological neurogenic trajectory—wherein neural progenitor cells (NPCs) undergo a final division before committing to neuronal fate—we evaluated SOX2 and DCX.

Our primary aim at this stage was to capture the broad lineage transition from stemness (SOX2) to early neuronal differentiation (DCX) induced rapidly upon MEIS1 depletion, rather than to dissect fine subtype shifts within specific intermediate progenitor sub-populations

5. Organoid region selection and timing (Fig 3)

Reviewer comment: Control and KD groups show unequal cortical thickness, biasing comparison; need GFP+ cell distribution by zone; unclear if time point is 1 week or 10 days.

Response: We sincerely thank the reviewer for pointing out this critical aspect regarding regional selection and potential analytical bias resulting from differences in cortical thickness between groups. To ensure that our data analysis is objective, robust, and free from selection bias, we first eliminated arbitrary region selection, and we strictly applied quantitative criteria for selecting cortical units (rosettes/cortical walls) for analysis. Regions were included based on structural integrity and clearly distinguishable apical-basal polarity, rather than overall tissue thickness. This ensured that functionally comparable, well-formed cortical structures were evaluated across both Control and KD conditions. We consider SOX2+ cells as VZ and DCX+ cells as non-VZ, with the combined total of SOX2+ and DCX+ cells accounting for over 80% of GFP-positive cells (Fig. 3C, D). At least three

rosettes per organoid were counted across multiple sections to obtain a single value for each organoid, and more than three organoids were analyzed. However, we retained only the data presented in Fig. 3B and are unable to reanalyze it for relative zone binning. We apologize for the inconvenience.

Accordingly, we have updated the text to include the relevant information.
page 5, line 130-134
page 5, line 135
page 19, line 465-467

6. Fig 4 quality and marker issues

Reviewer comment: Poor image quality (misaligned VZ/SVZ lines); larger sample size needed; PH3 mislabeled as proliferation marker (it's mitotic); neuron counts should be assessed later (E17.5).

Response: We appreciate this important point. According to the reviewer's suggestion, we performed additional experiments to observe the prolonged effects of *MEIS1* overexpression in mice. We found that *MEIS1* overexpression by in utero electroporation at E18 significantly increased the number of both CTIP2-positive cells (deep-layer neurons marker) and SATB2-positive cells (upper-layer neurons marker). These findings suggest that MEIS1 contributes to increase the neuronal populations in each layer.

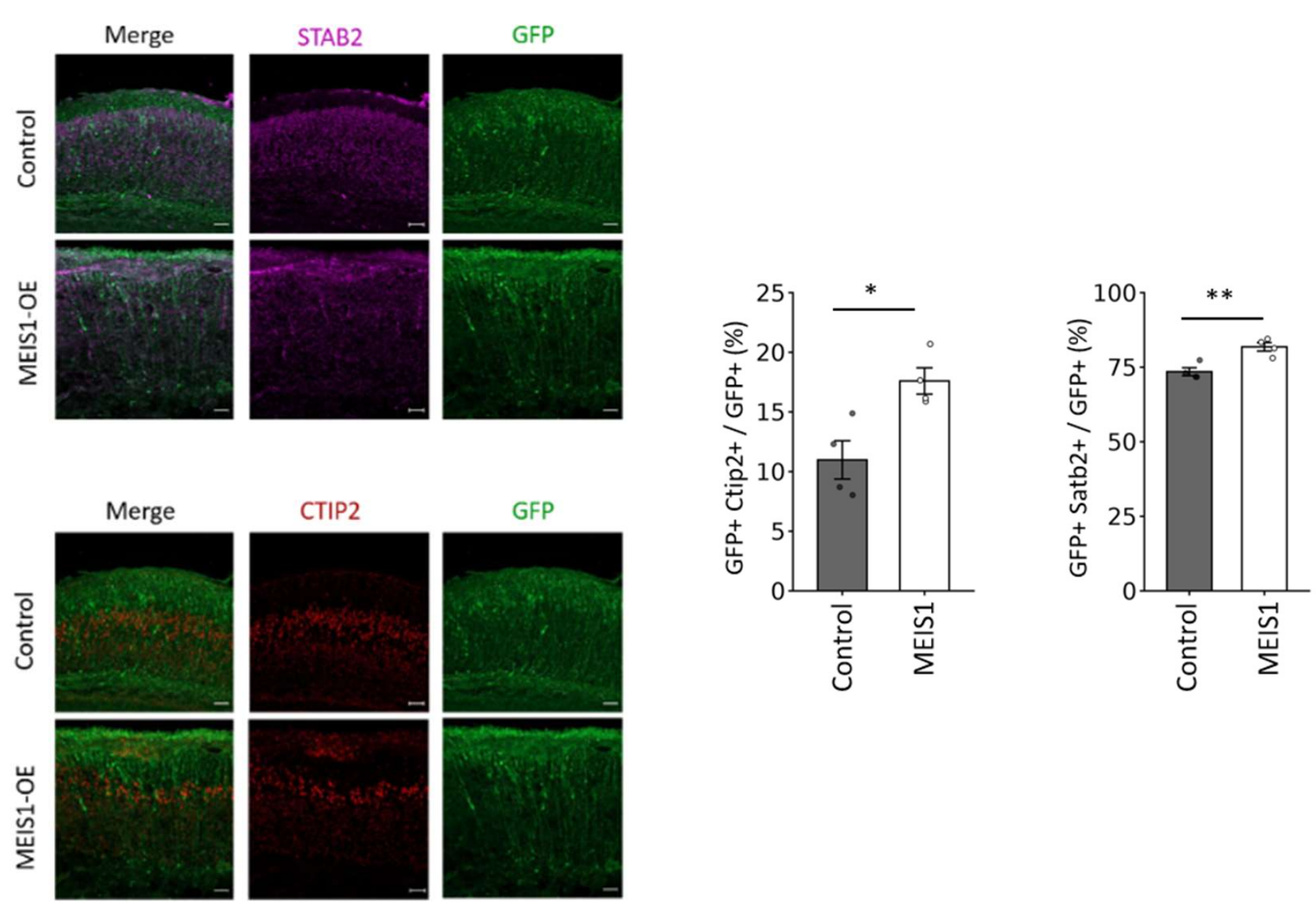

Accordingly, we have updated the text to include the relevant information.
page 6, lines 150–153, Fig. 4G-J

7. Endogenous MEIS1 protein levels

Reviewer comment: Physiological MEIS1 protein expression should be assessed in both mouse embryonic brain and human organoids.

Response: We thank the reviewer for pointing out the importance of clarifying physiological MEIS1 expression levels across these model systems.

Regarding the mouse embryonic brain, the physiological expression of Meis1 in the developing cerebral cortex has been documented in established literature (Isogai et al., 2022). Although we are confident about the high expression of MEIS1 in human NPCs, as demonstrated by our ChIP-seq pull-down data, we failed to detect its signal in human brain organoids. Sorry for this inconvenience.

8. NPC model rationale and organoid discrepancy

Reviewer comment: Unclear why NPC culture was used alongside organoids; EdU results contradict between NPC and organoid experiments — needs explanation.

Response: We sincerely thank the reviewer for this insightful comment. We agree that a clearer temporal model and direct cell-cycle analysis are essential to fully reconcile the observed phenotypes between 2D NPCs (AF22) and 3D cortical organoids. To directly address this point, we have newly quantified the S-phase duration in AF22 NPCs. We found that MEIS1 knockdown significantly extended the S-phase duration (Fig. S1). This new result directly demonstrates that *MEIS1* plays a crucial role in proper cell-cycle kinetics. Regarding the seemingly contradictory phenotypes between 2D monolayer cultures and 3D cortical organoids, we reason that these outcomes reflect fundamental differences in their cellular environments. In 2D monolayer cultures (AF22): Under pure, homotypic, and proliferation-saturated conditions, the extended S Phase and impaired cell-cycle kinetics lead to a straightforward reduction in the overall fraction of EdU-positive cells. In 3D cortical organoids: In a complex multicellular niche, cell-cycle progression is intimately coupled with fate-specification signals. In this context, the acute S-phase delay/stalling caused by *MEIS1* depletion may trigger an accelerated cell-cycle exit, which initially manifests as a transient accumulation of EdU-labeled cells, followed by premature differentiation and ultimate depletion of the progenitor pool.

We used NPC cultures in parallel with organoids because NPC cultures allow us to focus specifically

on neural stem/progenitor cells and thereby better highlight the role of MEIS1 in this cell population. In contrast, organoids contain multiple cell types and developmental stages, making it more difficult to assess the direct effects of MEIS1 on NPCs.

Accordingly, we have updated the text to include the relevant information.
page 7, lines 170–174, Fig. S1
page 12, lines 291–305

9. Fig 8 formatting inconsistency

Reviewer comment: Labeling style (Left/Right) differs from other figures; should be made consistent.

Response: We apologize for the oversight and inconsistency in the panel labeling of Fig. 8. In accordance with the reviewer's suggestion, we have completely revised Fig. 8 to unify its format and style with all other figures. Specifically, we removed the "(A) Left; Right" notations and assigned individual panel labels (A–D) to each image and corresponding quantification plot. The main text and figure legends have also been updated accordingly to reflect these consistent panel designations.

10. Regarding the connection to cholesterol synthesis

Reviewer comment: More data are needed to connect MEIS1, DHCR7, cholesterol synthesis and cilia morphology. The current writing in the results and discussion sounds very chunky and random. With the sequencing date, the connection between MEIS1 and DHCR7 is justified. The authors should perform additional experiments focusing on the functions of MEIS1 and DHCR7, and on cholesterol synthesis. Why one particular disorder-related phenotype - cilia length was chosen to study needs more justification as well. If the authors aim to report that DHCR7, cholesterol synthesis, and cilia morphology are mechanisms by which MEIS1 contributes to brain development, they should be examined in all three model systems and in the two species used in this study.

Response: We sincerely thank the reviewer for this insightful and constructive feedback regarding the logical flow, experimental depth, and rationale connecting MEIS1, DHCR7, cholesterol biosynthesis, and primary cilia morphology.

Primary cilia serve as an essential signaling hub in neural progenitor cells (NPCs), coordinating crucial pathways (such as Sonic Hedgehog signaling) that strictly govern NPC proliferation and differentiation. Biologically, *DHCR7* is well-established as a key terminal enzyme in the Kandutsch-Russell pathway of cholesterol biosynthesis, and primary cilia membranes are exceptionally enriched in cholesterol-rich microdomains (lipid rafts) that are vital for ciliary structure and function (Emmer

et al., 2010; Suzuki et al., 2020). Furthermore, mutations in DHCR7 cause Smith-Lemli-Opitz syndrome (SLOS), a neurodevelopmental disorder characterized by ciliary defects and microcephaly. These lines of evidence provided a strong biological rationale for testing whether MEIS1–DHCR7-dependent cholesterol regulation impacts ciliary integrity.

As the reviewer correctly pointed out, primary cilia assembly/disassembly is intimately coupled with cell-cycle kinetics and exit. Our gain-of-function experiments demonstrated that DHCR7 overexpression effectively rescues both the shortened primary cilia length and the impaired NPC proliferation induced by *MEIS1* depletion (Fig. 8B). This dual rescue strongly suggests a functional integration downstream of *MEIS1*, wherein *MEIS1* supports NPC proliferative capacity, at least in part, by maintaining ciliary integrity via transcriptional regulation of DHCR7.

To address the reviewer's concern regarding the narrative flow, we have revised the Discussion sections. We now explicitly articulate the biological rationale for investigating primary cilia and acknowledge alternative possibilities. Specifically, we have noted in the Discussion that the observed ciliary shortening may represent a secondary effect rather than a direct primary driver.

Accordingly, we have updated the text to include the relevant information.
page 13, line 320-326
page 14, line 342-352

11. Cilia length measurement method

Reviewer comment: Static 2D imaging is inadequate for ciliary length/morphology; 3D reconstruction or EM imaging needed for accurate quantification

Response: We appreciate this important point. While 3D reconstruction or EM imaging can provide more detailed assessments of ciliary morphology, ciliary length measurements based on 2D images are also widely used in the field. To minimize potential bias arising from cilium orientation, we quantified more than 60 cilia per condition and evaluated the overall trend across the population. Therefore, we believe that our analysis provides a reliable assessment of the differences in ciliary length.

12. Discussion depth

Reviewer comment: Should discuss MEIS1 function in brain development, cholesterol biosynthesis in broader context, species differences in cholesterol metabolism, and prior DHCR7 findings.

Response: We appreciate this important point. Particularly in the context of brain development, loss or deficiency of *DHCR7* has been shown to induce premature neurogenesis and deplete the cortical precursor pool in both murine and human neural progenitors (Tomita et al., 2022). This phenotype closely parallels our finding that MEIS1 KD—and the accompanying downregulation of DHCR7—drives human NPCs toward premature differentiation and pool exhaustion, further corroborating the critical role of the MEIS1–DHCR7 axis in cortical progenitor maintenance.

Accordingly, we have updated the text to include the relevant information.
page 13, line 320-326
page 14, line 342-352

13. Suggestion changing the title

Reviewer comment: Overall, the findings in the present study are more on the fundamental side, with less focus on the evolutionary expansion of the human brain. I would suggest that the authors choose a different title for the manuscript that better summarizes the role of MEIS1 in NPC biology, rather than the role of human-specific ncRNA in cortical expansion, especially since the study does not include any cross-species manipulative experiment addressing cortical expansion.

Response: According to the reviewer comments, we have changed the title to "Human-biased *MEIS1* expression by a promoter non-coding RNA regulates neural stem cell proliferation"

Minor comments

1. "in vivo" terminology

Reviewer comment: "... in vivo mouse brain" should be "... in mouse brain in vivo"

Response: We thank the reviewer for these helpful stylistic and grammatical corrections. We have updated the phrasing throughout the manuscript, changing "... in vivo mouse brain" to "... in mouse brain in vivo" as recommended (page 6, line 142-143).

2. Correction of typographical error

Reviewer comment: Typos including: TBR22

Response: We thank the reviewer for carefully reading our manuscript and catching these typographical errors. We have corrected "TBR22" to "Tbr2" in the Fig. 4 legend.

3. Correction of Fig. 7 legend (A)

Reviewer comment: Fig. 7 legend (A) needs to be corrected.

Response: We thank the reviewer for pointing this out. We have corrected the description in Fig. 7 legend (A).

4. Incomplete sentence

Reviewer comment: Page 8, second paragraph, sentence 1 is incomplete

Response: We thank the reviewer for bringing this grammatical oversight to our attention. We have corrected the sentence on page 9, line 215 to clearly state the methodology:
"To identify the primary targets of MEIS1, we performed MEIS1-targeted ChIP-seq."

Reviewer #3

1. bRG claim lacks data

Reviewer comment: bRG-like characteristics" claim in Introduction is unsupported—bRGs are never quantified or characterized. Need marker analysis (e.g., HOPX) or morphological criteria, or remove the claim

Response: We agree with the reviewer that PAX6+/PH3+ positivity and decreased Tbr2+ proportion alone are insufficient to definitively establish basal radial glia (bRG) identity without cell morphology analysis or specific bRG markers such as phospho-vimentin.
Rather than claiming a specific induction of bRG identity, we have softened our statements to focus on the broader effect of MEIS1 on NPC proliferation. Specifically, we have removed direct references to bRG identification in the mouse cortex and revised the manuscript to describe these cells as proliferating mouse NPCs and SVZ progenitors. The revised text emphasizes that *MEIS1* expression promotes the expansion and maintenance of the overall NPC pool without explicitly claiming the acquisition of bRG-specific cellular features.

Accordingly, we have updated the text to include the relevant information.
page 4, line 102-104

2. No validation of knockdown efficiency

Reviewer comment: *MEIS1* knockdown efficiency is not validated; need qRT-PCR, Western blot, or immunofluorescence evidence

Response: We would like to clarify that the knockdown efficiency of *MEIS1* has indeed been

validated and presented in Fig. 5B.

Specifically, Fig. 5B demonstrates a significant reduction in *MEIS1* expression following shRNA-mediated knockdown (sh-MEIS1), confirming the high efficiency and specificity of our targeting constructs.

3. Contradictory EdU results across models

Reviewer comment: EdU results are inconsistent: MEIS1 KD increases EdU in organoids but decreases it in NPCs, while MEIS1 OE increases proliferation in mouse. These need to be reconciled.

Response: We sincerely thank the reviewer for this insightful comment. We agree that a clearer temporal model and direct cell-cycle analysis are essential to fully reconcile the observed phenotypes between 2D NPCs (AF22) and 3D cortical organoids.To directly address this point, we have newly quantified the S-phase duration in AF22 NPCs. We found that *MEIS1* knockdown significantly extended the S-phase duration (Fig. S1). This new result directly demonstrates that *MEIS1* plays a crucial role in proper cell-cycle kinetics. Regarding the seemingly contradictory phenotypes between 2D monolayer cultures and 3D cortical organoids, we reason that these outcomes reflect fundamental differences in their cellular environments. In 2D monolayer cultures (AF22): Under pure, homotypic, and proliferation-saturated conditions, the extended S Phase and impaired cell-cycle kinetics lead to a straightforward reduction in the overall fraction of EdU-positive cells. In 3D cortical organoids: In a complex multicellular niche, cell-cycle progression is intimately coupled with fate-specification signals. In this context, the acute S-phase delay/stalling caused by MEIS1 depletion may trigger an accelerated cell-cycle exit, which initially manifests as a transient accumulation of EdU-labeled cells, followed by premature differentiation and ultimate depletion of the progenitor pool.

Accordingly, we have updated the text to include the relevant information.
page 7, lines 169–173, Fig. S1
page 12, lines 288–302

4. Accelerated cell-cycle claim unsupported

Reviewer comment: "Accelerated cell cycle transition" claim lacks direct cell-cycle length measurement. Unclear which NPC subtype (aRG/bIP/bRG) is affected; why SOX2 staining is absent outside VZ; what evidence supports acceleration.

Response: We sincerely thank the reviewer for this insightful comment. We agree that a clearer

temporal model and direct cell-cycle analysis are essential to fully reconcile the observed phenotypes between 2D NPCs (AF22) and 3D cortical organoids. To directly address this point, we have newly quantified the S-phase duration in AF22 NPCs. We found that *MEIS1* knockdown significantly extended the S-phase duration (Fig. S1). This new result directly demonstrates that *MEIS1* plays a crucial role in proper cell-cycle kinetics. Regarding the seemingly contradictory phenotypes between 2D monolayer cultures and 3D cortical organoids, we reason that these outcomes reflect fundamental differences in their cellular environments. In 2D monolayer cultures (AF22): Under pure, homotypic, and proliferation-saturated conditions, the extended S Phase and impaired cell-cycle kinetics lead to a straightforward reduction in the overall fraction of EdU-positive cells.In 3D cortical organoids: In a complex multicellular niche, cell-cycle progression is intimately coupled with fate-specification signals. In this context, the acute S-phase delay/stalling caused by MEIS1 depletion may trigger an accelerated cell-cycle exit, which initially manifests as a transient accumulation of EdU-labeled cells, followed by premature differentiation and ultimate depletion of the progenitor pool.

Accordingly, we have updated the text to include the relevant information.
page 7, lines 169–173, Fig. S1
page 12, lines 288–302

5. Effect on neuronal populations
Reviewer comment: Tbr2+ progenitors decrease (Fig 4E-F) — is neuron number also affected?

Response: We appreciate this important point. According to the reviewer's suggestion, we performed additional experiments to observe the prolonged effects of MEIS1 overexpression in mice. We found that *MEIS1* overexpression by in utero electroporation at E18 significantly increased the number of both CTIP2-positive cells (deep-layer neurons marker) and SATB2-positive cells (upper-layer neurons marker). These findings suggest that MEIS1 contributes to increase the neuronal populations in each layer.

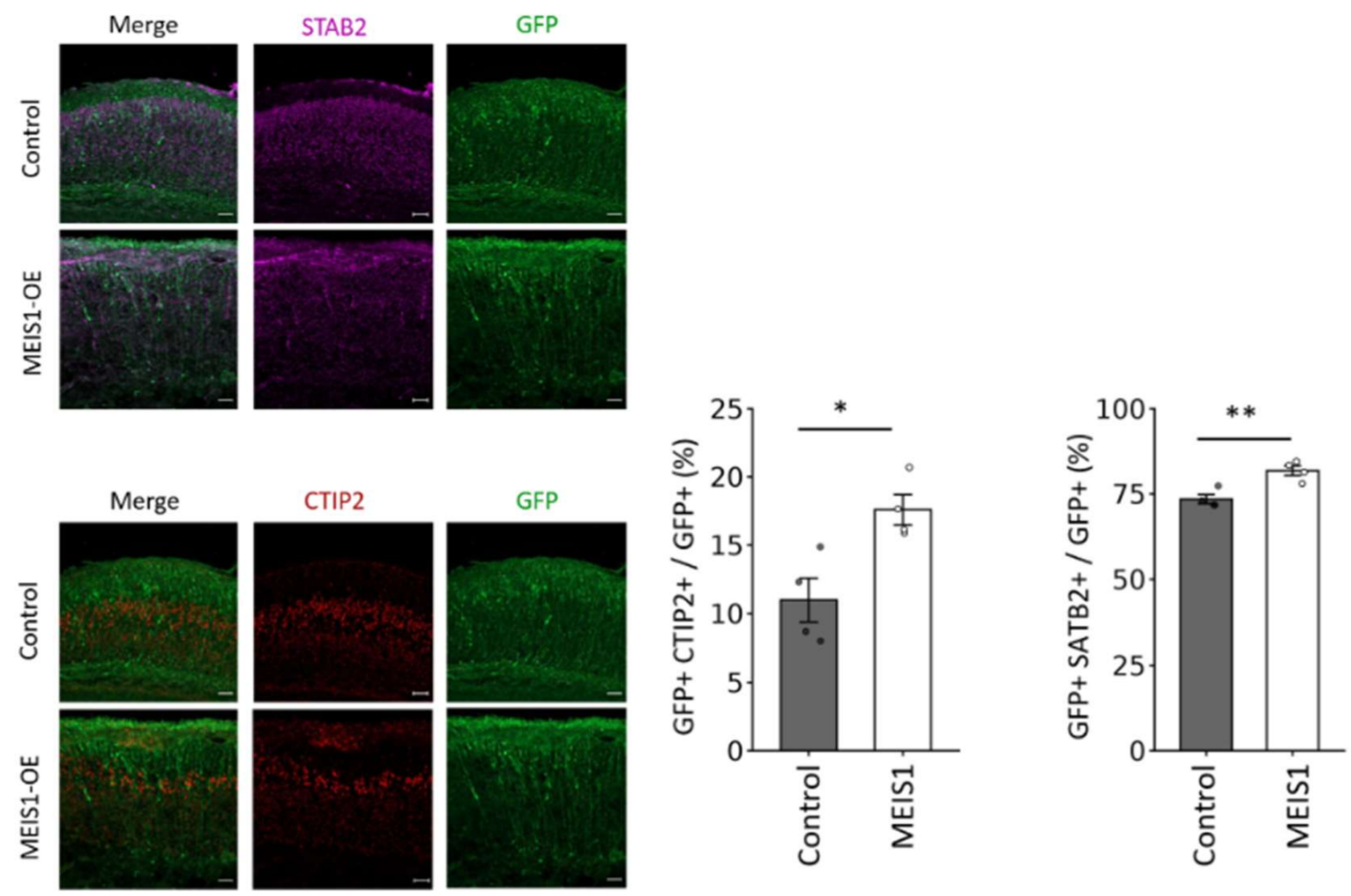

Accordingly, we have updated the text to include the relevant information.
page 6, lines 150–153, fig. 4G-J

6. Which NPC subtype maintains stemness / bRG-like?

Reviewer comment: High MEIS1 maintaining stemness — which NPC subtype is affected? Tbr2 decrease + PH3 increase in VZ — does this indicate bRG-like cells? Need HOPX or similar marker analysis.

Response: We agree with the reviewer that PAX6+/PH3+ positivity and decreased Tbr2+ proportion alone are insufficient to definitively establish basal radial glia (bRG) identity without cell morphology analysis or specific bRG markers such as phospho-vimentin.
Rather than claiming a specific induction of bRG identity, we have softened our statements to focus on the broader effect of *MEIS1* on NPC proliferation. Specifically, we have removed direct references to bRG identification in the mouse cortex and revised the manuscript to describe these cells as proliferating mouse NPCs and SVZ progenitors. The revised text emphasizes that MEIS1 expression promotes the expansion and maintenance of the overall NPC pool without explicitly claiming the acquisition of bRG-specific cellular features.

Accordingly, we have updated the text to include the relevant information.
page 4, line 102-104

7. "Human-specific" claim needs cross-species data

Reviewer comment: pancMEIS1 being "human-specific" is insufficiently supported by mouse-only comparison; other primates should be analyzed.

Response: We sincerely thank the reviewer for this constructive and insightful comment. We agree that restricting comparative evidence to human versus mouse is insufficient to firmly establish a broader evolutionary conclusion regarding human-specific traits.

To address this limitation and thoroughly test the evolutionary conservation of *pancMEIS1,* we newly analyzed RNA-seq data derived from chimpanzee NPCs. As shown in the newly added Integrative Genomics Viewer (IGV) snapshot shown below, pancMEIS1 expression is clearly detected in human NPCs, whereas it is completely absent in both mouse and chimpanzee NSCs.

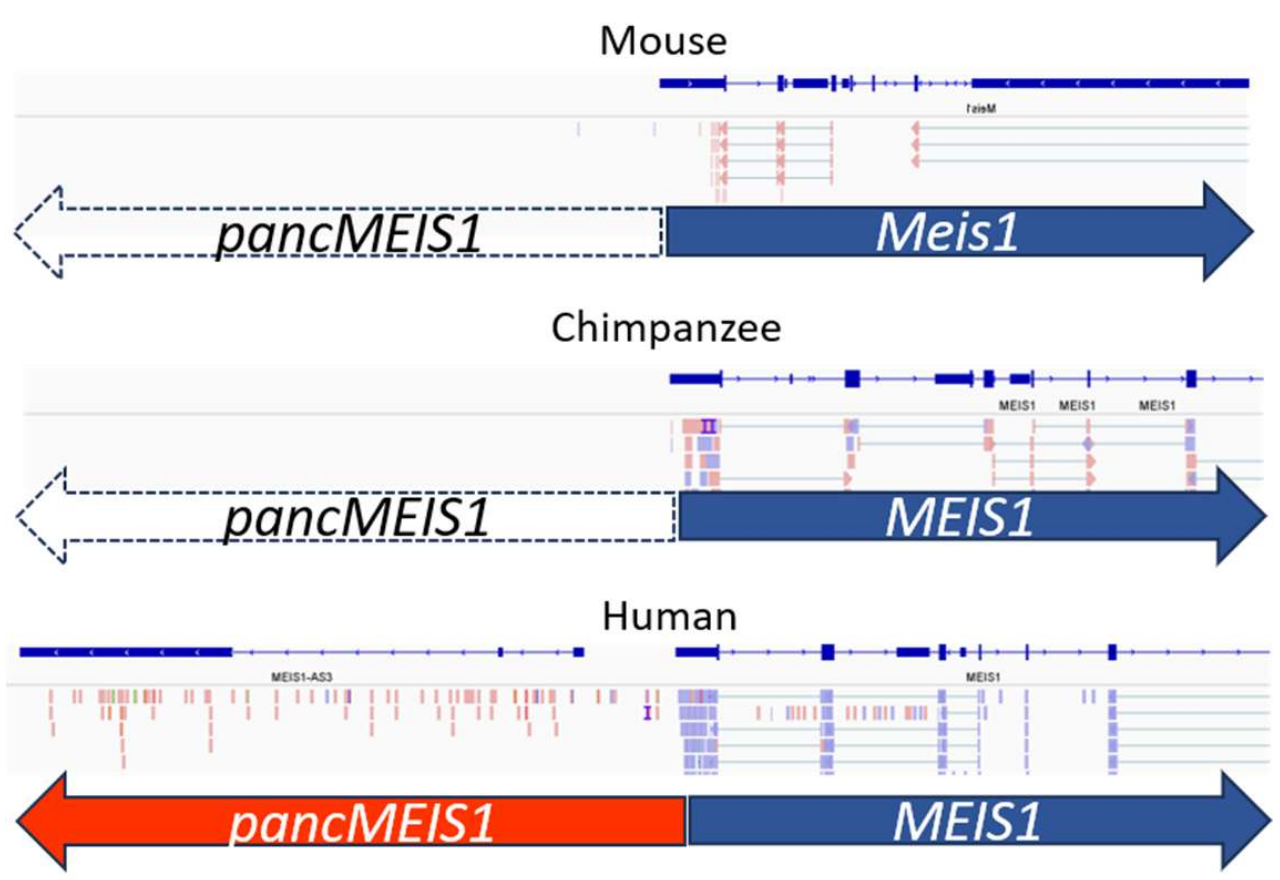

Accordingly, we have updated the text to include the relevant information.

page 7, lines 184–187, fig. 6A

8. Modest MEIS1-AS knockdown efficiency

Reviewer comment: *MEIS1-AS* KD reduces *MEIS1* only ~20% — unclear if phenotypes are MEIS1-mediated or MEIS1-independent effects of the ncRNA.

Response: We appreciate this important point. We additionally performed rescue experiments by overexpressing *MEIS1* in *pancMEIS1* KD cells. *MEIS1* overexpression significantly rescued the proliferation defect induced by *pancMEIS1* depletion, demonstrating that *pancMEIS1* regulates cell proliferation in a MEIS1-dependent manner. Moreover, analysis using public strand-specific DRIP-seq identified an R-loop peak at the *MEIS1* promoter locus (SRR11185387 and SRR11185388: Yan

et al., Cell Rep. 32, 107870, 2020), raising the possibility that *pancMEIS1* functions in cis at the *MEIS1* promoter to promote *MEIS1* expression through an R-loop-dependent regulatory mechanism.

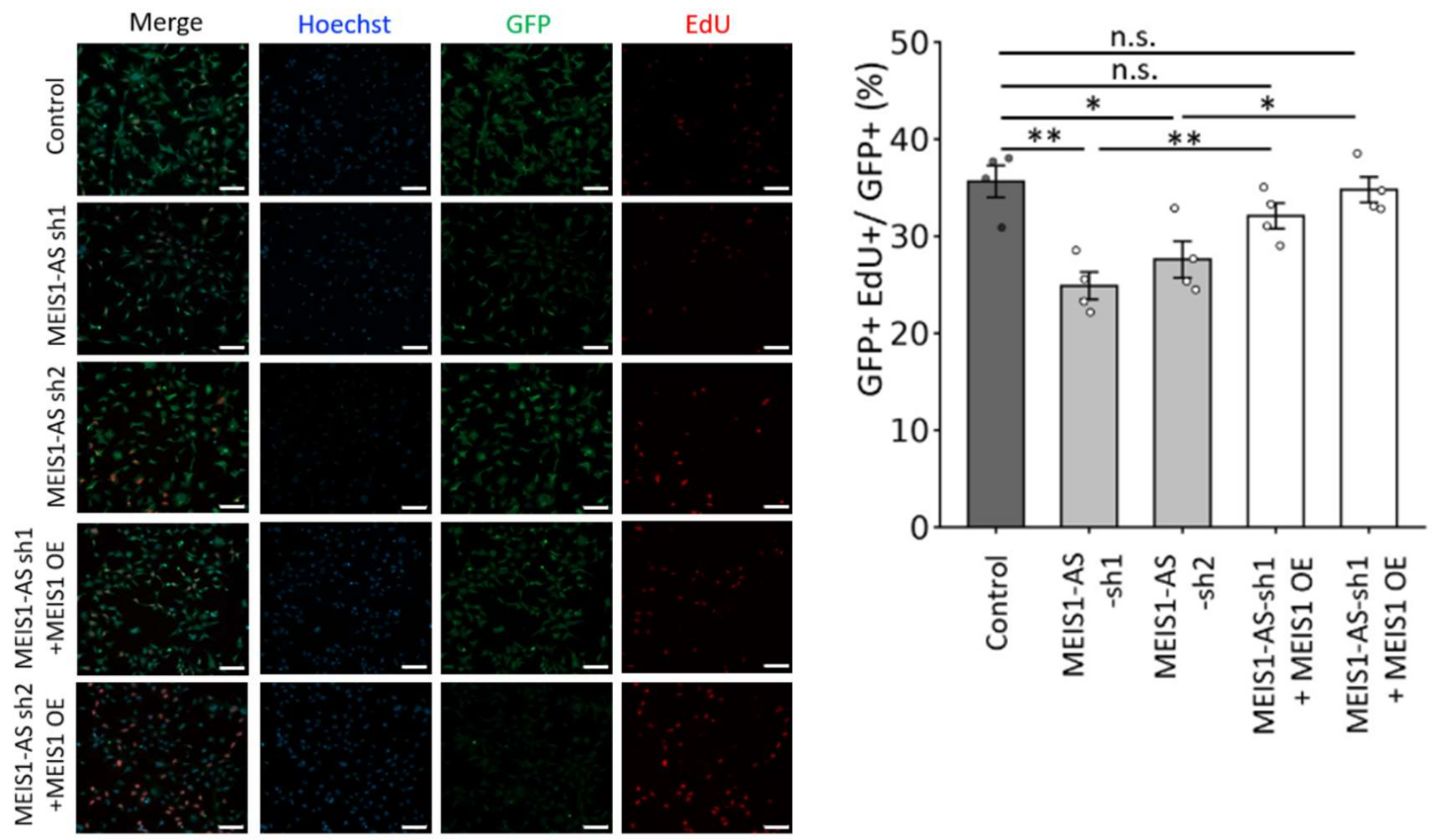

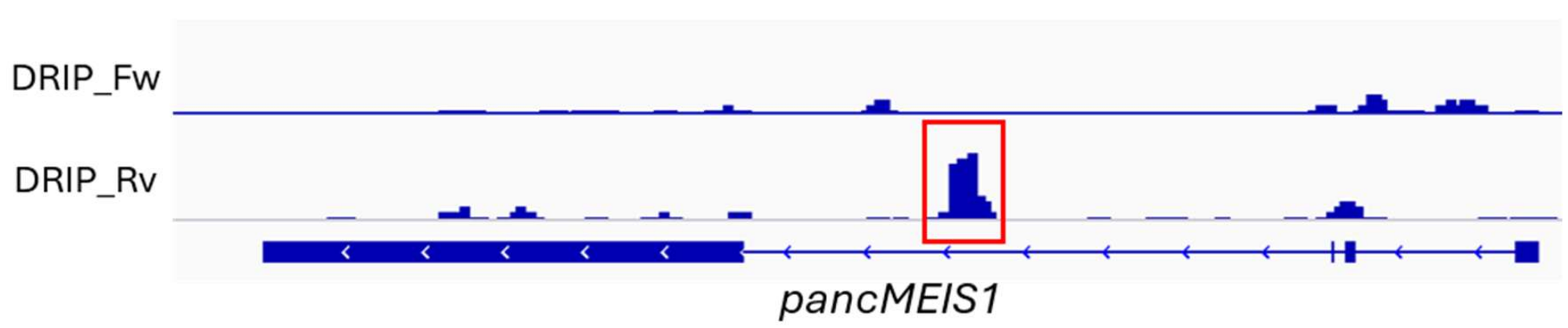

Accordingly, we have updated the text to include the relevant information.

page 8, lines 196–201, Fig. 6E and F

page 8, lines 187-189, Fig. S2

9. ectopic expression of *MEIS1-AS* in embryonic mouse

Reviewer comment: It would be informative to test whether ectopic expression of MEIS1-AS in embryonic mouse increases MEIS1 expression.

Response: We thank the reviewer for this insightful suggestion regarding the ectopic expression of *MEIS1-AS* in the embryonic mouse cortex. In this study, we prioritized human-derived cell models (human NPCs) to establish the direct functional and regulatory interplay between *MEIS1-AS* and *MEIS1* within a human molecular background. While performing in vivo ectopic expression in embryonic mice represents an exciting future direction to explore its systemic impact, our current

investigation was centered on validating these core cell-intrinsic mechanisms in human models.

To address the reviewer's point, we have updated the Discussion section to explicitly mention the potential of in vivo ectopic expression assays in animal models as an important direction for future research.

Accordingly, we have updated the text to include the relevant information.
page 15, line 360-364

10. cutoff of RNA-seq analysis

Reviewer comment: For RNA-seq analysis, the authors use a relatively lenient cutoff (log2 fold change > 0.3). More stringent thresholds may help identify more robust and biologically relevant candidates.

Response: We thank the reviewer for this constructive suggestion regarding the threshold selection for our RNA-seq analysis. To rigorously justify our threshold selection, we evaluated multiple logFC cutoff values(log2FC=0.3, 0.5, 1.0) for downregulated genes and performed pathway enrichment analyses across each threshold set. By tracking the statistical significance (minimum p value) of the top enriched pathways, we observed that applying a threshold of log2FC > 0.3 yielded the strongest pathway enrichment (the highest -log10 (p-value)) compared to more stringent cutoffs (left figure). Applying higher fold-change thresholds compromised the statistical power of downstream pathway enrichment analyses, resulting in higher p-values for key biological pathways. At log2FC > 0.3, we identified 1,280 differentially expressed genes (DEGs)—a well-balanced sample size that effectively captures functionally relevant pathways without introducing excessive background noise. Based on these quantitative optimization results, we confirmed that log2FC > 0.3 is the most biologically informative and robust threshold for our dataset.

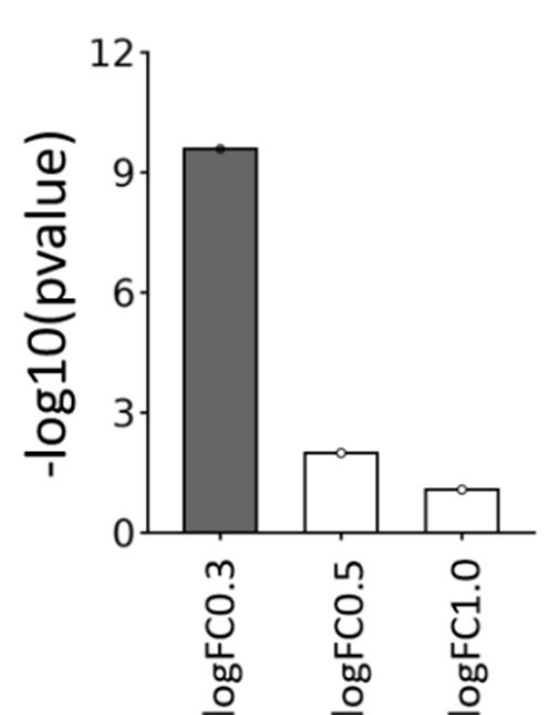

Minor Comments:

1. **y-axis label in Fig. 1A**

Reviewer comment: Fig. 1A lacks units. Please specify whether the data represent TPM, relative expression, or arbitrary units.

Response: We thank the reviewer for pointing this out. The data in Fig. 1A were normalized by TPM. We have now explicitly indicated "TPM" on the y-axis of Fig. 1A.

2. **Developmental stage of the dataset from Fietz et al. (2012)**

Reviewer comment: The dataset from Fietz et al. (2012) includes multiple fetal stages. Please indicate which developmental stage(s) are shown.

Response: We thank the reviewer for requesting clarification regarding the developmental stages of the dataset from Fietz et al. (2012). To secure sufficient sequencing depth for reliable downstream comparison, we pooled multiple samples for each species to perform the analysis as a representative single composite dataset (N = 1). Specifically, for the human dataset, we merged six samples across 13 to 16 p.c.w.. For the mouse dataset, we merged five samples at E14.5.

3. **A discrepancy in Fig. 2**

Reviewer comment: There is a discrepancy in Fig. 2: the text states that organoids were derived from *MEIS1* KD iPSCs, whereas the schematic suggests lentiviral knockdown in organoids. Please clarify.

Response: We appreciate the reviewer catching this inconsistency between the main text and the schematic in Fig. 2. To clarify, *MEIS1* KD was performed via lentiviral transduction directly on brain organoids, as correctly depicted in the schematic, rather than generating organoids from pre-established *MEIS1* KD iPSCs. We sincerely apologize for the confusion caused by the description in the text.

Accordingly, we have updated the text to include the relevant information.
page 5, line 123-124

4. **Pax6 data are not shown in Fig. 3**

Reviewer comment: In Fig. 3, the authors mention changes in Pax6+ cells, but Pax6 data are not shown.

Response: We thank the reviewer for catching this error. The reference to "Pax6+" in the text was a typographical error; the data presented in Fig. 3 actually correspond to SOX2+ cells. We have removed all mentions of "Pax6" from this section and corrected the text to accurately refer to SOX2+ cells in the revised manuscript.

Accordingly, we have updated the text to include the relevant information.
page 5, line 135
page 11, line 269

5. **Correction of pH3 terminology**
Reviewer comment: Phospho-histone H3 is described as a proliferation marker; more precisely, it is a mitotic marker.

Response: We thank the reviewer for this accurate point. We have updated the manuscript accordingly, changing the description of Phospho-histone H3 from "proliferation marker" to "mitotic marker".

Accordingly, we have updated the text to include the relevant information.
page 6 line 145

6. **Citation of Fig. 5A in the main text**
Reviewer comment: Fig. 5A is not referenced in the text.

Response: We thank the reviewer for bringing this oversight to our attention. We have now cited Fig. 5A in the revised Results section where the experimental design for *MEIS1* KD is described.

Accordingly, we have updated the text to include the relevant information.
page 6, line 158

7. **Incomplete sentence**
Reviewer comment: The sentence "To identify the primary targets of MEIS1, we performed MEIS1-targeted ChIP-seq using" is incomplete.

Response: We thank the reviewer for bringing this grammatical oversight to our attention. We have corrected the sentence on page 9, line 215 to clearly state the methodology:
"To identify the primary targets of MEIS1, we performed MEIS1-targeted ChIP-seq."

8. **Correction regarding mouse basal progenitors in the SVZ**
Reviewer comment: The statement "In mouse context, BPs in SVZ usually have low proliferation because largely they are not bRGs any more and have begun to differentiate into neurons" is

inaccurate. In mice, basal progenitors are primarily bIPs and have not yet differentiated into neurons.

Comment: We thank the reviewer for this critical correction. We agree that basal progenitors (BPs) in the mouse SVZ are predominantly basal intermediate progenitors (bIPs)—which undergo limited neurogenic divisions rather than having already differentiated into neurons.

In accordance with this point, we have revised the text in the Discussion section to accurately describe mouse BPs as predominantly neurogenic bIPs with limited proliferative capacity, avoiding the inaccurate claim that they have already begun to differentiate into neurons.

Accordingly, we have updated the text to include the relevant information.
page 11-12, line 278-282

September 9, 2026

RE: Life Science Alliance Manuscript #LSA-2026-03708-TR

Prof. Takuya Imamura
Hiroshima University
Graduate School of Integrated Sciences for Life
Kagamiyama 1-3-1
Higashi-Hiroshima, Hiroshima 7398526
Japan

Dear Dr. Imamura,

Thank you for submitting your revised manuscript entitled "Human-biased MEIS1 expression by a promoter non-coding RNA regulates neural stem cell proliferation".

Your manuscript was evaluated by all the original reviewers whose comments are appended below. As you will note, Reviewers 1 and 3 have acknowledged your revised manuscript with Reviewer 3 positively commenting on it. That said, Reviewer 1 has noted insufficient details in the methods and need to rephrase certain results and conclusions. Reviewer 2 has listed concerns around unresolved confounding data in case of Figures 5D, 2, and 3.

In line with the overall evaluation of Reviewers 1 and 3, we would be happy to publish your paper in Life Science Alliance pending resolution of the above mentioned points from the reviewers (without new experiments) and final revisions necessary to meet our formatting guidelines. Please also include a rebuttal with point-by-point responses to the reviewers' comments.

MANUSCRIPT ORGANIZATION AND FORMATTING:

To avoid unnecessary delays in the acceptance and publication of your paper, please read the following information carefully. Full guidelines are available on our Instructions for Authors page, https://www.life-science-alliance.org/authors

Submission of a paper that does not conform to Life Science Alliance guidelines will delay the acceptance of your manuscript.

-We encourage you to use another terminology instead of 'human-specific' in your abstract (lines 29 and 35), consistent with changes made to title.
-Please provide details on iPSCs in the section on organoid generation, "Briefly, feeder-free iPSCs (provided by Dr. Hideyuki Okano) were fed daily .. "
-Kindly expand on details for imaging: microscope name, objective name and numerical aperture/magnification
-Please provide antibody concentration for information in line 583.
-Please define TPM in the legend for Figure 1
-Please provide scale bar information for Figure 4G, I
-Please upload all figure files as individual ones, including the supplementary figure files; all figure legends should only appear in the main manuscript file.
-Please add the X and Bluesky handles of your host institute/organization, as well as your own, and/or one of the authors, in our system.
-Please rename "Data accessibility" to "Data Availability" and "Competing interests" to "Conflict of interest".
-Please use the [10 author names et al.] format in your references (i.e., limit the author names to the first 10).
-Please add your main and supplementary figure legends to the main manuscript text after the references section.
-Please upload your main figures as single files; these will be displayed in-line in the HTML version of your paper, so please provide them as single-page files (Figures); we do not have a limit on the number of main figures, and these can be split if necessary for space.
-Please add an Author Contributions section to your main manuscript text.
-Please be sure that the authorship listing and order is correct

We encourage our authors to provide original source data, particularly uncropped/-processed electrophoretic blots and spreadsheets for the main figures of the manuscript. If you would like to add source data, we would welcome one PDF/Excel-file per figure for this information. These files will be linked as supplementary "Source Data" files.

It is Life Science Alliance policy that if requested, original data images must be made available to the editors. Failure to provide original images upon request will result in unavoidable delays in publication. Please ensure that you have access to all original data images prior to final submission.

If you are planning a press release on your work, please inform us immediately to allow informing our production team and scheduling a release date.

We welcome submissions of potential cover images for the issue of LSA in which your work would appear. If you have high quality images associated with this work, please feel free to email these, with a caption, to the journal office.

LSA encourages authors to provide a 30-60 second video where the study is briefly explained. These videos will be appear embedded with the manuscript online at Life Science Alliance, and on social media to promote the published paper and authors (for examples, see https://docs.google.com/document/d/1-UWCfbE4pGcDdcgzcmiuJl2XMBJnxKYeqRvLLrLSo8s/edit?usp=sharing). Corresponding or first-authors are welcome to submit the video. Please submit only one video per manuscript. The video can be emailed to contact@life-science-alliance.org

FINAL FILES:

To upload the final version of your manuscript, please log in to your account: https://lsa.msubmit.net/cgi-bin/main.plex
You will be guided to complete the submission of your revised manuscript and to fill in all necessary information. Please get in touch in case you do not know or remember your login name.

The following items are required for acceptance.

-- An editable version of the final text (.DOC or .DOCX) is needed for copyediting (no PDFs).

-- High-resolution figure, supplementary figure and video files uploaded as individual files: See our detailed guidelines for preparing your production-ready images, https://www.life-science-alliance.org/authors

-- Summary blurb (enter in submission system): A short text summarizing in a single sentence the study (max. 200 characters including spaces). This text is used in conjunction with the titles of papers, hence should be informative and complementary to the title. It should describe the context and significance of the findings for a general readership; it should be written in the present tense and refer to the work in the third person. Author names should not be mentioned.

The license to publish form must be signed before your manuscript can be sent to production. A link to the license to publish form will be available to the corresponding author only. Please take a moment to check your funder requirements.

Reviews, decision letters, and point-by-point responses associated with peer-review at Life Science Alliance will be published, alongside the manuscript. If you do want to opt out of having the reviewer reports and your point-by-point responses displayed, please let us know immediately.

Thank you for your attention to these final processing requirements. Please revise and format the manuscript and upload materials as soon as you are able.

Thank you for this interesting contribution to the literature. We look forward to publishing your paper in Life Science Alliance.

Sincerely,

Sarita Hebbar, PhD
Scientific Editor
Life Science Alliance
http://www.lsajournal.org

Reviewer #1 (Comments to the Authors (Required)):

The authors have addressed all my comments. Few minor comments remain, mainly because the authors didn't correctly report the details of the newly added data in the Methods and some main text phrasings should be reworded.

1. The authors have now added chimp RNA-seq data and show that the signal corresponding to pancMEIS1 is detected only in human NPCs but not in chimp or mouse NPCs. This is a useful addition, but the claim still requires caution as lack of expression in one dataset might also reflect technical differences in library preparation, sequencing depth, or differences in expression linked to the timepoint and/or tissue analyzed. Therefore, I suggest more conservative wording for this result in the main text.

2. The addition of SATB2 and CTIP2 measurements is very helpful. However, the methods section currently does not report the details of this experiment, as the methods section on in utero electroporation states that animals were sacrificed at E15.5 without

describing the E18.5 cohort; SATB2 is both in the figure and figure legend often misspelled as STAB2, antibodies used for SATB2 and CTIP2 are not mentioned in the Methods, etc... The authors must carefully go through the newly added experiments and properly report everything in the methods section.

3. Similarly, I dont'see description of the newly added DRIP-seq analysis in the Methods.

Reviewer #2 (Comments to the Authors (Required)):

The authors have improved the writing to avoid overstatement and toned down the conclusion. However, many of the major concerns raised earlier are still not addressed adequately.

In the decision letter, the editor stated that two common concerns shared by all three reviewers: "We highlight the reviewers common concerns on incomplete descriptions, and contradictory results with MEIS1 knockdown in organoids versus NPCs
-We concur with the reviewers that you must explain this contradiction (Reviewer 1, point 4, Reviewer 2, point 8, Reviewer 3, point 3).
-We also agree that descriptions must be more in-depth and accurate (eg. include other markers, NPC sub-types; Reviewer 2, points 4-6, Reviewer 3, points 4-6)."

The contradiction between NPC and organoid KD results is not addressed. The authors performed one additional experiment to measure S-phase length in NPCs and reported that it is extended upon MEIS1 KD. Theoretically, an extended S phase should allow more NPCs in culture to incorporate EdU and increase the overall abundance of EdU+ cells. This is not the case in Fig.5D, where the proportion of EdU+ cells is reduced.

The authors did not provide in-depth, accurate descriptions of the other markers suggested by all reviewers, nor did they explain the discrepancy between Fig. 2, which shows increased EdU+ cells, and Fig. 3, which shows decreased Sox2+ cells. Without further analysis, the claimed "Accelerated neuronal differentiation" phenotype is unsupported, which significantly affects the paper's overall conclusion.

Because many of the suggested revision analyses were not performed, the authors, in my opinion, missed an opportunity to revise and strengthen the manuscript, and many of the major concerns raised earlier still remain.

Reviewer #3 (Comments to the Authors (Required)):

I thank the authors for carefully addressing all of my previous comments and concerns. The revised manuscript has been substantially improved, and I have no further comments or suggestions. I am satisfied with the revisions and support publication of the manuscript in its current form.

Reply to Reviewer #1

1. pancMEIS1 signal: species-specific or technical difference

Reviewer comment: the absence of *pancMEIS1* signal in chimp/mouse NPCs may reflect species-specific expression, but could also reflect technical differences (library prep, sequencing depth) or timepoint/tissue variation. Please soften the wording accordingly.

Response: We thank the reviewer for this important point. To adopt more conservative wording, we have replaced "human-specific" with "human-biased" throughout the manuscript.

2. Methods section incomplete for SATB2/CTIP2 experiments

Reviewer comment: The addition of SATB2 and CTIP2 data is helpful, but the Methods section is incomplete: the E18.5 cohort is not described (only E15.5 is mentioned), "SATB2" is misspelled as "STAB2" in the figure and legend, and the antibodies used are not listed. Please carefully update the Methods to fully report these new experiments.

Response: We thank the reviewer for pointing out these important oversights. We have thoroughly reviewed the newly added experimental data and updated the manuscript as follows:

-We have revised the *in utero* electroporation protocol in the Methods section to explicitly include the description for the E18.5 analysis cohort alongside E15.5

-Detailed antibody information for SATB2 and CTIP2 have been added to the Methods section of Immunohistochemistry.

- Typographical Correction: We have corrected all instances of the spelling error "STAB2" to "SATB2" across the main text, figures, and figure legends.

3. Methods section incomplete for DRIP-seq analysis experiments

Reviewer comment: Similarly, I don't see description of the newly added DRIP-seq analysis in the Methods.

Response: We apologize for this omission. We have now added a dedicated subsection titled "DRIP-seq data analysis" to the Methods section, describing the full pipeline including data acquisition, quality trimming, genome alignment, PCR duplicate removal, strand separation using Samtools, and bigWig track generation (page 23, line 567-577)

Reply to Reviewer #2

1. **S-phase/EdU contradiction in NPCs**

Reviewer comment: We concur with the reviewers that you must explain this contradiction.

Response: We interpret the observed prolongation of S phase following *MEIS1* knockdown as reflecting a cell-autonomous mechanism, whereas *in vivo*, the outcome reflects the integrated influence of the niche microenvironment. We elaborate on this point in the Discussion:

(In the Result section: page 7, line 163 – line 165)
However, in human brain organoids, *MEIS1* KD transiently increased the proportion of EdU+ proliferating cells. This observation likely reflected a mixed microenvironment containing both proliferative and differentiation signals.

(In the Discussion: page 12, line 301 – page 13, 311)
In 2D monolayer cultures, where cells are maintained under homotypic, proliferation-saturated conditions, MEIS1 depletion leads to a significant reduction in EdU incorporation, demonstrating that MEIS1 plays a primary, cell-intrinsic role in promoting cell proliferation and sustaining cell cycle kinetics. Conversely, 3D cortical organoids represent a complex heterotypic niche where proliferative cues, cell-cell interactions, and fate-specification signals dynamically interplay. In such a multicellular architecture, cell cycle regulation is intimately coupled to neuronal differentiation and lineage progression (Lange et al., 2009). Thus, the transient EdU elevation after *MEIS1* KD in organoids does not contradict the role of MEIS1 in promoting proliferation; Rather, as supported by the quantitative determination showing an extended S-phase duration upon *MEIS1* KD (Fig. S1), we believe that MEIS1 is fundamentally required for proper NPC proliferation.

2. **Fig. 2–3 discrepancy undermines differentiation claim**

Reviewer comment: We also agree that descriptions must be more in-depth and accurate (eg. include other markers, NPC sub-types.

Response: We thank the reviewer for this important point. Our primary objective in this study was not to detail fine shifts within specific intermediate progenitor subtypes, but rather to capture the overarching lineage transition from uncommitted stemness to early neuronal differentiation. To address the Reviewer's point and enrich our discussion, we have updated the Discussion section to emphasize that future studies incorporating markers such as NGN1, HOPX, and TBR2 to trace specific

cell subtypes will be essential to fully elucidate the cell-type-specific responses (page 11, line 278 – page 12, 282).

September 18, 2026

RE: Life Science Alliance Manuscript #LSA-2026-03708-TRR

Prof. Takuya Imamura
Hiroshima University
Graduate School of Integrated Sciences for Life
Kagamiyama 1-3-1
Higashi-Hiroshima, Hiroshima 7398526
Japan

Dear Dr. Imamura,

Thank you for submitting your Research Article entitled "Human-biased MEIS1 expression by a promoter non-coding RNA regulates neural stem cell proliferation". It is a pleasure to let you know that your manuscript is now accepted for publication in Life Science Alliance. Congratulations on this interesting work.

The final published version of your manuscript will be deposited by us to PubMed Central upon publication.

Your manuscript will now progress through copyediting and proofing. It is journal policy that authors provide original data upon request.

Your article will publish open access upon publication under a CC-BY license.

Reviews, decision letters, and point-by-point responses associated with peer-review at Life Science Alliance will be published, alongside the manuscript. If you do want to opt out of having the reviewer reports and your point-by-point responses displayed, please let us know immediately.

IMPORTANT: If you will be unreachable at any time, please provide us with the email address of an alternate author. Failure to respond to routine queries may lead to unavoidable delays in publication.

Scheduling details will be available from our production department. You will receive proofs shortly before the publication date. Only essential corrections can be made at the proof stage so if there are any minor final changes you wish to make to the manuscript, please let the journal office know now.

DISTRIBUTION OF MATERIALS:
Authors are required to distribute freely any materials used in experiments published in Life Science Alliance. Authors are encouraged to deposit materials used in their studies to the appropriate repositories for distribution to researchers.

You can contact the journal office with any questions, contact@life-science-alliance.org

Again, congratulations on a very nice paper. I hope you found the review process to be constructive and are pleased with how the manuscript was handled editorially. We look forward to future exciting submissions from your lab.

Sincerely,

Sarita Hebbar, PhD
Scientific Editor
Life Science Alliance
http://www.lsajournal.org
